# Tetravalent Terbium Chelates: Stability Enhancement and Property Tuning

**DOI:** 10.1021/prechem.3c00065

**Published:** 2023-10-02

**Authors:** Tianjiao Xue, You-Song Ding, Xue-Lian Jiang, Lizhi Tao, Jun Li, Zhiping Zheng

**Affiliations:** † Department of Chemistry, Southern University of Science and Technology, Shenzhen, Guangdong 518055, China; ‡ Department of Chemistry and Engineering Research Center of Advanced Rare-Earth Materials of Ministry of Education, Tsinghua University, Beijing 100084, China; § Key University Laboratory of Rare Earth Chemistry of Guangdong, Southern University of Science and Technology, Shenzhen, Guangdong 518055, China

**Keywords:** Tetravalent Terbium Ion, Chelating Ligand, Formal Potential, Stability, Magnetic Property

## Abstract

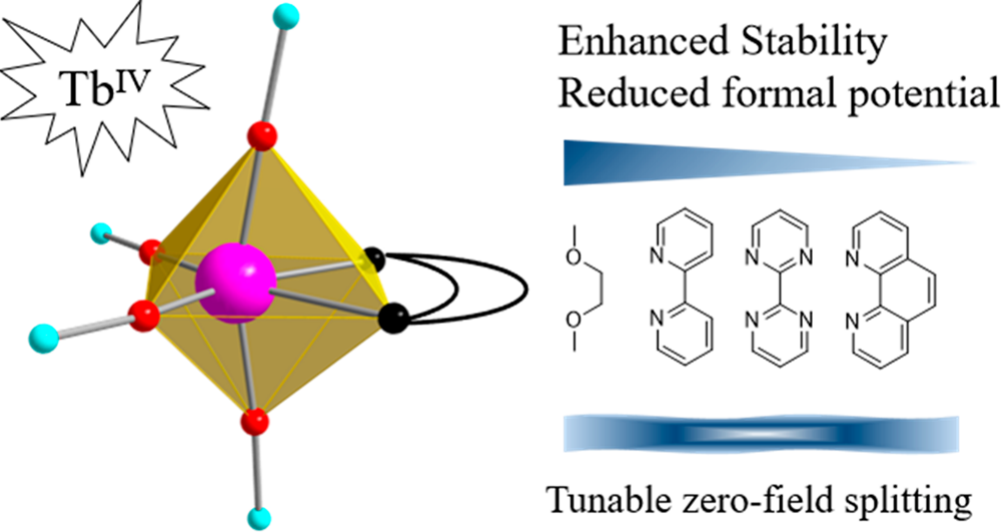

Coordination chemistry of rare-earth elements has been dominated by the +3 oxidation state. Complexes with higher-valence lanthanide ions are synthetically challenging but are of fundamental research interest and significance as advanced molecular materials. Herein, four tetravalent terbium complexes (**2**–**5**) of the common formula [Tb(OSiPh_3_)_4_L] (L = ethylene glycol dimethyl ether (DME), 2,2^’^-bipyridine (bpy), 2,2^’^-bipyrimidine (bpym), and 1,10-phenanthroline (phen)) are reported. Crystallographic analyses reveal in each of these complexes a hexacoordinate Tb(IV) ion situated in a distorted octahedral coordination environment formed by four triphenylsiloxido ligands and a bidentate chelating ligand. The use of chelating ligands enhances the stability of the resulting complexes over their THF solvate precursor. More significantly, the aromatic N-chelating ligands have been found to tune effectively the electronic structures of the complexes, as evidenced by the sizable potential shifts observed for the quasi-reversible redox Tb(IV/III) process and by the changes in their absorption spectra. The experimental findings are augmented with quantum theoretical calculations in which the ligand π-donation to the 5*d* orbitals of the Tb(IV) center is found to be primarily responsible for stability enhancement and the corresponding changes of physical properties observed. Magnetic measurements and results from electron paramagnetic resonance studies produced small absolute values of zero-field splittings of these complexes, ranging from 0.1071(22) to 1.1484(112) cm^–1^ and comparable to the values reported for analogous Tb(IV) complexes.

## Introduction

Complexes with their metal ions in unusual oxidation states are of interest for fundamental research and applications of practical significance. For example, high-valence iron species play a major role in oxidative reactions in nature,^1^ while actinide ions in their unconventionally high oxidation states enable their effective separation from a gamut of coexisting rare earth ions.^2,3^ In this context, the coordination chemistry of the lanthanide (Ln) ions in unconventional oxidation states is of particular relevance and of high interest to researchers interested in theoretical and computational chemistry, synthetic and structural chemistry, physical properties and chemical reactivity, and materials applications of the lanthanides. However, the chemistry of the lanthanides is dominated by the +3 oxidation state, with the few exceptions of Ce(IV), Sm(II), Eu(II), and Yb(II) being reasonably readily accessible.^4−10^ With the pioneering work by Lappert and co-workers^11^ and the following efforts by Evans, Mazzanti, Meyer, Long, and others, remarkable progress in the chemistry of Ln(II) ions has been achieved.^12−27^ The isolation of divalent complexes of all lanthanide elements was completed in 2013,^23^ some of which display intriguing physical properties with potential applications as high-performing single-molecule magnets^26^ and molecular spin qubits.^27^ In stark contrast, the chemistry of high-valence lanthanide ions has lagged far behind due primarily to the lack of synthetic methods or reagents and the notorious air/moisture sensitivity or reactivity of such high-valence species. In this context, the formation of Pr(V) species in the gas phase and in a solid noble-gas matrix is noteworthy.^28,29^ It is also of note that the Tb(IV) ion in the solid was identified more than 70 years ago,^30,31^ but complexes of tetravalent lanthanide other than Ce(IV) were unknown until very recently. It is the efforts led independently by Mazzanti^32−35^ and La Pierre^36−38^ that have broken new ground in the chemistry of tetravalent lanthanide complexes.^39^

**Scheme 1:**
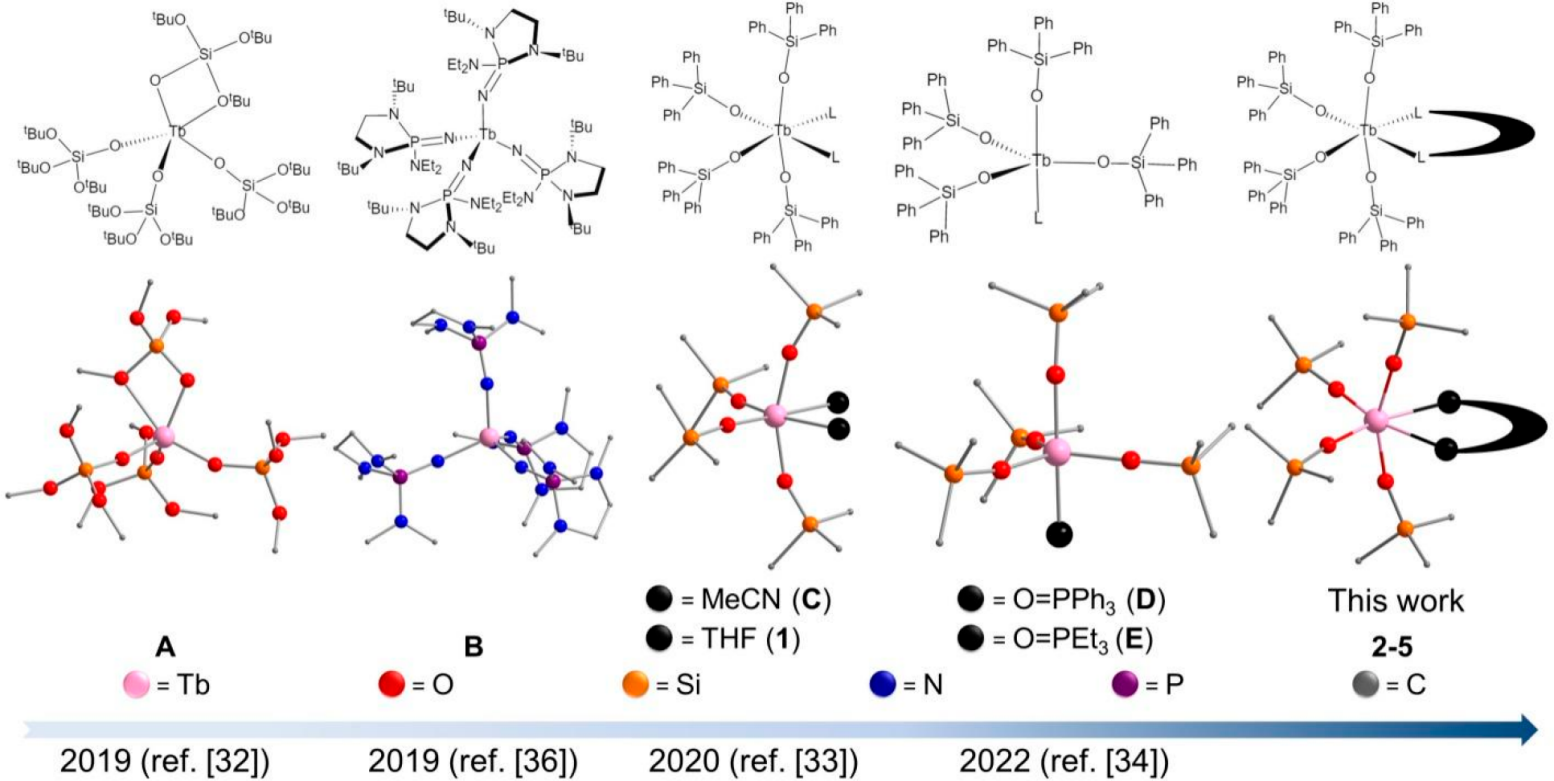
Structures of the Tb(IV) Complex in the Literature The rightmost one represents the generic structure of the chelates in this work.

A number of representative Tb(IV) complexes are collected in time order as shown in Scheme 1, the first being [Tb(OSi(O^*t*^Bu)_3_)_4_] (**A**) reported by Mazzanti and co-workers with its pentacoordinate Tb(IV) center situated in a coordination sphere formed by four OSi(O^*t*^Bu)^−^ ligands.^32^ La Pierre et al. reported [Tb(NP(1,2-bis-^*t*^Bu-diamidoethane)(NEt_2_))_4_] (**B**) stabilized by phosphinimine ligands (PN*) in the same year.^36,37^ Shortly after, Mazzanti and co-workers reported the first Pr(IV) complex [Pr(OSiPh_3_)_4_(MeCN)_2_]^35^ by adopting the same procedure for the preparation of its Tb(IV) congeners [Tb(OSiPh_3_)_4_(L)_2_] (L = CH_3_CN (**C**) or THF (**1**)) and [Tb(OSiPh_3_)_4_(L)] (L = O=PPh_3_ (**D**) or O=PEt_3_ (**E**)).^33,34^ Out of the small number of tetravalent lanthanide complexes, these of Pr(IV) and Tb(IV), featuring, respectively, 4*f*^1^ and 4*f*^7^ electron configurations, are magnetically attractive, both capable of providing a pure nuclear-spin environment and hyperfine coupling between nuclear and electronic spins. Potential applications as single-molecule magnets and molecular spin qubits can be envisioned.^40−44^ It is thus important to develop tetravalent Pr(IV) and Tb(IV) complexes with enhanced stability and to study their physicochemical properties with an eye on the aforementioned applications.

**Scheme 2:**
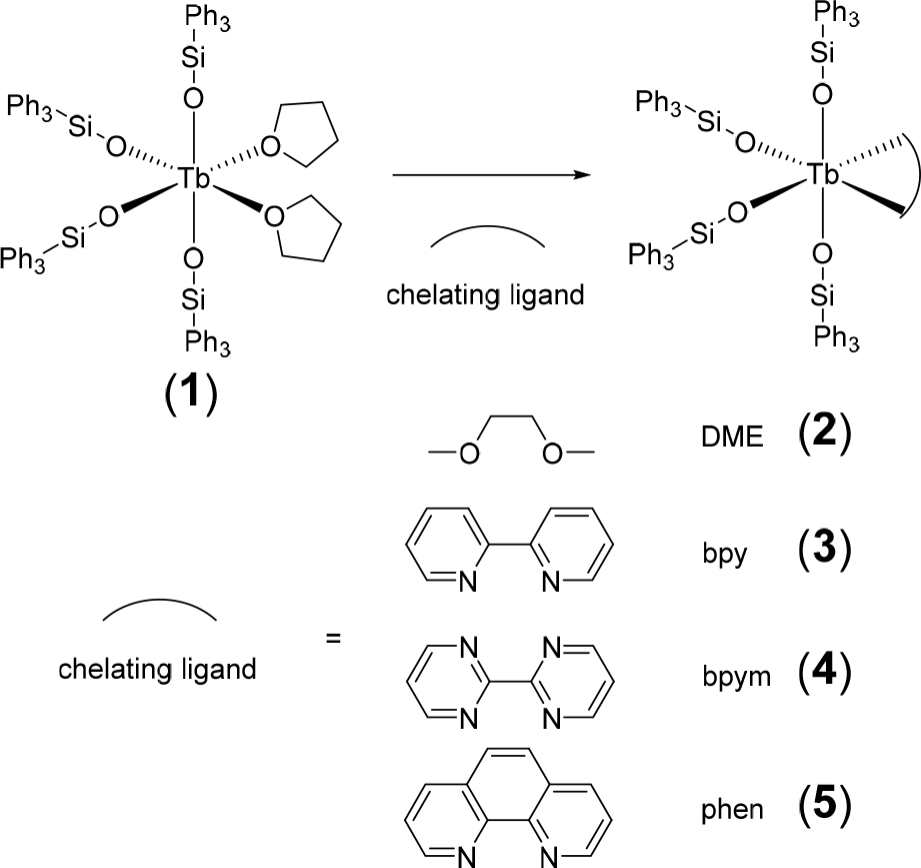
Syntheses of **2**–**5** by Ligand Exchange of Tb(OSiPh_3_)_4_(THF)_2_ (**1**) with the Chelating Ligands Shown

Ligand substitution was studied by Mazzanti and co-workers who found that the coordinated solvent molecules in [Tb(OSiPh_3_)_4_(L)_2_] (L = CH_3_CN or THF) can be replaced by phosphinoxide ligands,^33,34^ leading to the enhanced stability of the resulting complexes due to the strong π(O–Tb) interaction from phosphinoxide ligands. Inspired by these literature reports and in hopes of producing tetravalent lanthanide complexes with even further enhanced stability for property studies, we explore in this work the replacement of the coordinated tetrahydrofuran (THF) molecules in Tb(OSiPh_3_)_4_(THF)_2_ (**1**)—one of the early examples of Tb(IV) complexes—with a number of O- and N-chelating ligands. Specifically, the syntheses and crystal structures of four new tetravalent Tb(IV) complexes (**2**–**5**, Scheme 2) are reported, with each featuring four triphenylsiloxido ligands and a bidentate chelating ligand, including ethylene glycol dimethyl ether (DME), 2,2′-bipyridine (bpy), 2,2′-bipyrimidine (bpym), and 1,10-phenanthroline (phen). Property studies by cyclic voltammetry, absorption spectroscopy, and DFT calculations reveal enhanced stability of the chelates over the precursor complex with two coordinated THF molecules and collectively point to the stabilizing effect of the chelating ligands. Magnetic measurements and studies by electron paramagnetic resonance (EPR) spectroscopy indicate that the absolute values of zero-field splitting for these complexes are relatively small.

## Results and Discussion

### Syntheses and Structural Characterization

Complex **1** was obtained by adopting a literature procedure:^34^ Oxidizing the trivalent precursor Tb(OSiPh_3_)_3_(THF)_3_ (**Tb**^**Ph3**^)^45^ with [(C_6_H_4_Br)_3_N][SbCl_6_] in the presence of KOSiPh_3_, followed by recrystallization from THF at −30 °C, afforded the desired product. LiOSiPh_3_ or NaOSiPh_3_ can be used in place of KOSiPh_3_, but the desired tetravalent complex was not formed without this additional equivalent of siloxido to balance the extra positive charge of the newly generated Tb(IV).^33,34^ Complexes **2**–**5** were obtained by ligand exchange of **1** with its coordinated THF being replaced by DME, bpy, bpym, and phen, respectively (Scheme 2).

**Figure 1:**
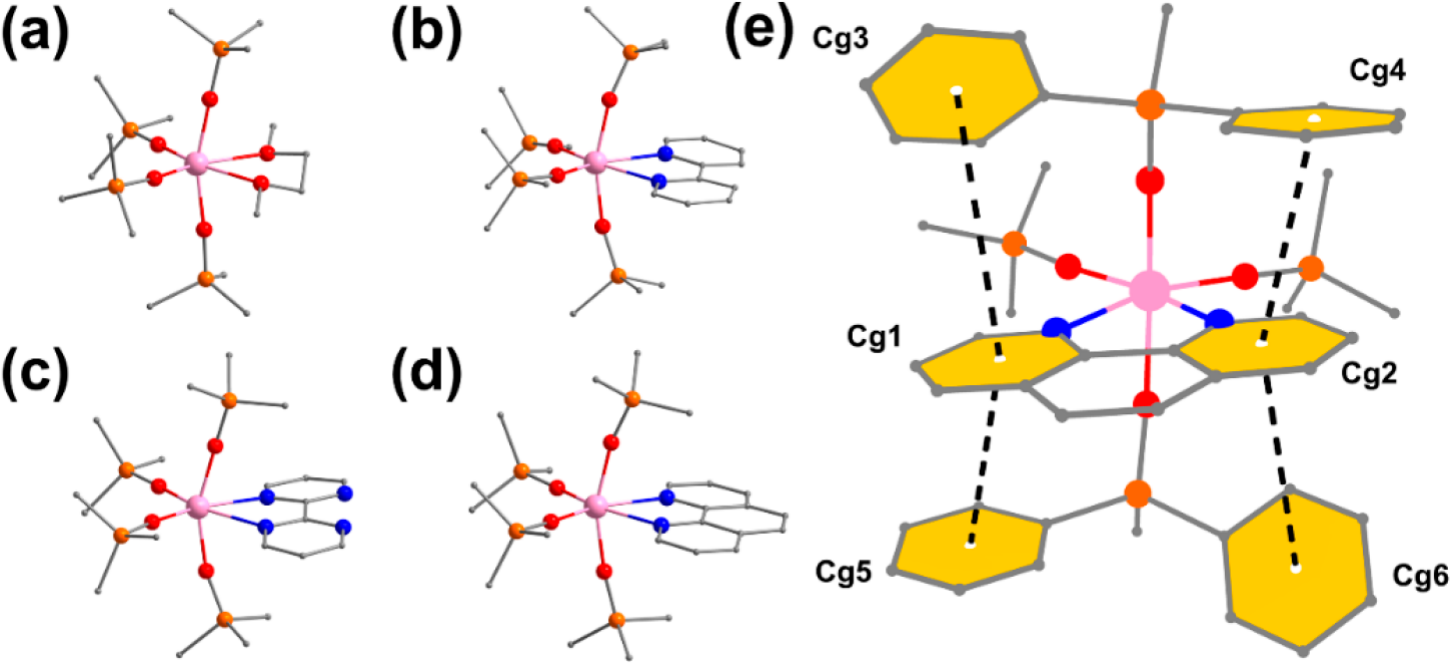
Stick-and-ball depiction of the crystal structures of (a) **2**, (b) **3**, (c) **4**, and (d) **5**, other atoms are omited for clarity; (e) π–π interactions between the phenyl groups of the axial siloxido ligands and the aromatic rings of the phen ligand in **5**; yellow hexagons highlight the aromatic groups involved (color code: Tb, pink; O, red; Si, orange; C, gray; N, blue).

The solid-state structures of the four new complexes were established by single-crystal X-ray diffraction studies (Table S1). As shown in Figure 1, each of the four new complexes features a hexacoordinated Tb(IV) ion situated in a distorted octahedral coordination sphere formed by four Ph_3_SiO^–^ ligands and a unique bidentate chelating ligand. Overall, these new complexes are structurally similar to the previously reported **1**(34) and [Tb(OSiPh_3_)_4_(CH_3_CN)_2_] (**C**)^33^ with two *cis*-disposed nonsiloxido ligands. Except for solvent molecules of recrystallization, no ions of any kind are present in the complete crystal structure, which is consistent with the complexes being electrically neutral. Bond lengths and angles of interest are summarized in Table 1. The Tb–O_siloxido_ bonds of the new complexes are comparable with these of the Tb(IV) complexes previously reported,^32−34^ but shorter than these of the trivalent precursor **Tb**^**Ph3**^. Ranging from 65.09(4)° to 66.87(14)°, the O–Tb–O or N–Tb–N angles associated with the chelating ligands in **2**–**5** are significantly smaller than the O_THF_–Tb–O_THF_ angle in **1** (86.10(12)°), due presumably to the enhanced rigidity of the bidentate chelating ligands. Correspondingly, the angles between the two siloxido ligands that are coplanar with the chelating ligands, ranging from 100.10(4)° to 108.13(10)°, are significantly larger than the corresponding angle of 96.02(11)° in **1** (Table S2). This scenario is entirely understandable as the less crowded coordination of the chelating ligand makes room for a more relaxed disposition of the coplanar siloxido ligands. The remaining two siloxido ligands are “steered away” due to steric repulsion by the equatorial siloxido ligands, resulting in a pronounced deviation of the axial coordination motif from linearity (157.14(12)° to 165.07(10)°) (Table S2). Two phenyl groups on each of the axial siloxido ligands are disposed in such a way that strong face-to-face π–π interactions are formed with the aromatic rings of the N-chelating ligand (Figures 1e, S1, S2; Tables S3–S5) The distortion of the coordination geometry from a perfect octahedron was estimated by continuous shape measures analysis^46^ to be 0.654, 1.041, 1.297, 1.330, and 1.185 for **1**–**5**, respectively (Table S6). Albeit small, such deformation of the coordination polyhedra can perturb the electronic structure of a lanthanide complex, leading to significant changes in magnetic properties.^47,48^

**Table 1:** Selected Bond Lengths (Å) and Angles (deg) of **Tb**^**Ph3**^ and **1**–**5**

	Tb–O_siloxido_	Tb–O/N (L)^a^	O/N (L)–Tb–O/N (L)
**Tb**^**Ph3**^	2.135(3)–2.145(3)	2.441(3)–2.484(4)	
**1**	2.043(2)–2.079(2)	2.400(2)	86.10(12)
**2**	2.032(3)–2.084(3)	2.439(3)–2.443(3)	66.81(11)
**3**	2.039(4)–2.094(4)	2.462(5)–2.473(4)	65.38(14)
**4**	2.027(1)–2.078(1)	2.497(1)–2.503(1)	65.09(4)
**5**	2.044(2)–2.085(2)	2.461(3)	66.87(14)

a O/N (L) indicates the coordinating atoms (O in **1** and **2;** N in **3**–**5** of the neutral ligand L).

### Cyclic Voltammetry

**Figure 2:**
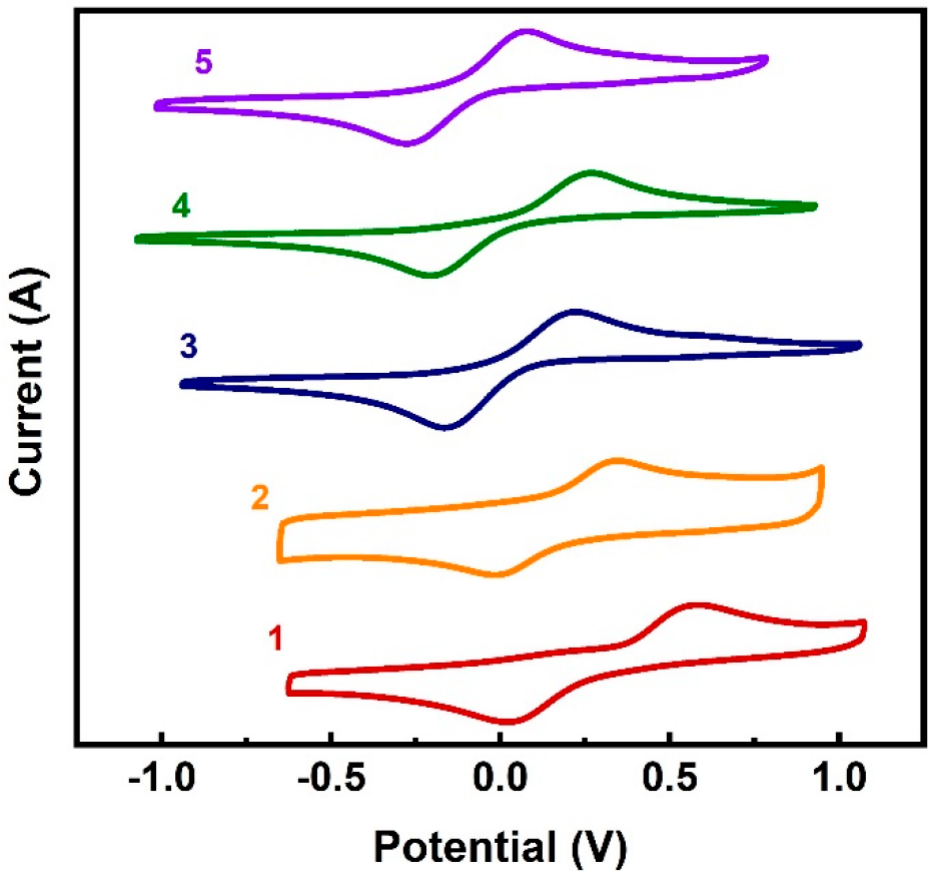
Cyclic voltammograms of **1**–**5** (1 mM in dichloromethane) with [N^*n*^Bu_4_][B(C_6_F_5_)_4_] (0.1 mM) as supporting electrolyte at a sweep rate of 500 mV s^–1^ vs Fc/Fc^+^.

Redox properties of the complexes were studied by cyclic voltammetry (CV). The voltammograms are each characterized by a single pair of redox events, exhibiting a quasi-reversible redox process. As shown by the data collected in Table 2 (Figure 2), the reduction (*E*_pc_) and oxidation (*E*_pa_) potentials both decrease upon chelation of the Tb(IV) center, with the peaks shifting from 0.020 and 0.582 V of **1** to −0.278 and 0.078 V for **5**, respectively. The *E*_pa_ of 0.078 V for **5** is the smallest among all Tb(IV) siloxido complexes, and is only higher than that of **B**, a tetravalent terbium complex with a ligand of strong π character.^32−34,36^

**Table 2:** Electrochemical Data for the Tb(III/IV) Peak Couple of **1**–**5** vs Fc/Fc^+^ (Fc = Ferrocene) in dichloromethane at a Sweep Rate of 500 mV s^–1^

Complex	*E*_pc_ (V)	*E*_pa_ (V)	*E*° (V)	Δ*E* (V)	*I*_pa_/*I*_pc_
**1**	0.020	0.582	0.301	0.562	0.894
**2**	–0.017	0.346	0.165	0.363	0.996
**3**	–0.164	0.223	0.030	0.387	0.823
**4**	–0.208	0.266	0.029	0.474	0.792
**5**	–0.278	0.078	–0.100	0.356	1.093

The peak separations (Δ*E*) for **2**–**5** is smaller than that of **1** by about 0.1–0.2 V, and even smaller than these of the other previously reported Tb(IV) complexes (Table 2),^32−34,36^ reflecting well the enhanced stability of the chelates during the redox process. The relationship between the peak current and the square root of the scan rate was found to be nearly linear, and the Δ*E* increased with increasing scan rate, suggesting that Tb(III/IV) redox reactions are diffusion-controlled. For **2**–**5**, the ratio of peak currents (*I*_pa_/*I*_pc_) ranges from 0.8 to 1.2, indicating that the reduced species were mostly reoxidized upon reversal of the scan direction, exhibiting good chemical reversibility (Figures S5–S12). In contrast, the peak current ratio of **1** varies more sensitively upon change of scan rate (Figures S3 and S4, Table S7). This result suggests that the redox process of **1** is more complex, possibly involving dissociation and recoordination of the THF ligands, a scenario corroborated by the large separation between the reduction and oxidation peaks observed for **1**.

The formal potential (*E*°), a good reflection of the relative thermodynamic stability of the Tb(IV) and Tb(III) redox states,^49−51^ ranges from −0.1 V of **5** to 0.301 V of **1** (Table 2). In other words, the use of chelating ligands is propitious to enhancing the thermodynamic stability of a complex, which is completely expected. Soundly supporting this conclusion is the shift of *E*° from 0.301 V for **1** to 0.165 V for **2** with the mere substitution of two coordinated THF molecules with a single DME ligand. It appears that introduction of a conjugated chelating ligand can further enhance the stability of the complex as reflected by the further reduced *E*°, to 0.030, 0.029, and −0.100 V for **3**–**5**, respectively (Table 2). Complex **5** emerged as the most thermodynamically stable among complexes **1**–**5**, probably owing to the additional conjugated phenyl ring relative to the 2,2′-bipyridine ligand.

### UV–Vis Spectra

**Figure 3:**
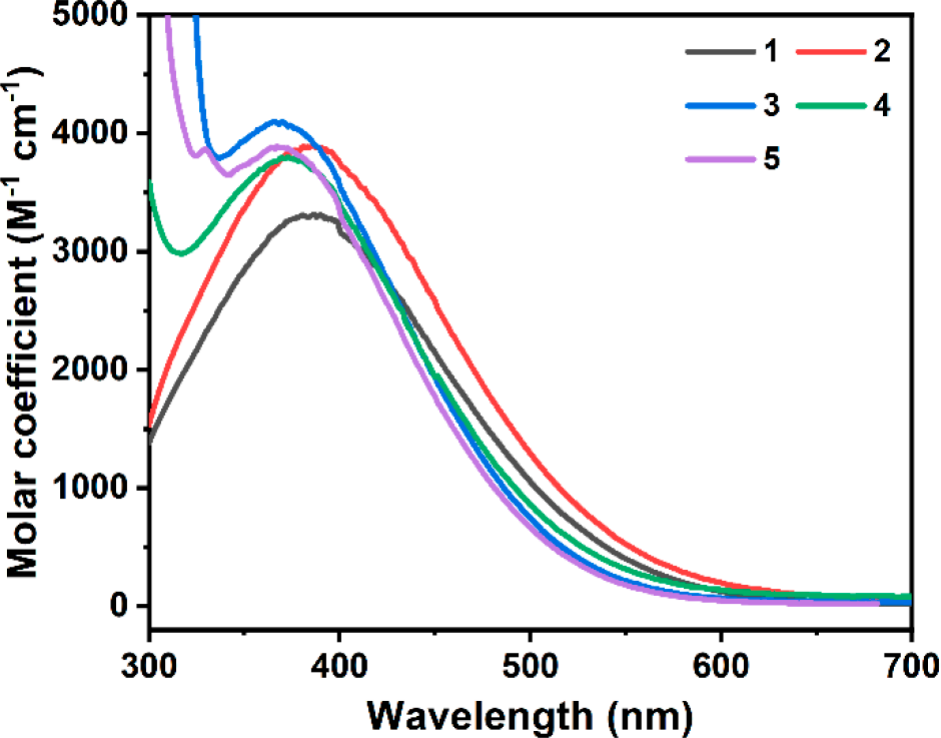
UV–vis absorption spectra of **1**–**5** in toluene at room temperature.

The UV–vis spectra of **1**–**5** were collected immediately following dissolution in toluene (Figure 3) and dichloromethane (Figure S13). The absorptions of **1** and **2** in toluene, spanning between 285 and 575 nm, show maxima at ca. 382 nm, while the absorptions of the N-chelates (**3**–**5**) covers the 320–550 nm range with maxima at ca. 371 nm. The spectra obtained with dichloromethane solutions are essentially the same as these with the toluene solutions with only a <5 nm shift of the absorption maxima (Table 3). The molar absorptions, ranging from 3300 to 4200 M^–1^ cm^–1^ for **1**–**5**, are comparable to the values reported for analogous Tb(IV) siloxido complexes^32−34^ and an electrochemically generated Tb(IV) species.^52,53^

**Table 3:** Experimental and TDDFT Results (nm) of the Absorption Maxima for **1**–**5**

Complexes	Dichloromethane	Toluene	Excitation energy	Assignments
**1**	382	385	388	LMCT
**2**	383	384	384	LMCT
**3**	370	370	378/373	LLCT/LMCT
**4**	374	373	377/375	LLCT/LMCT
**5**	370	370	376/374	LLCT/LMCT

To rationalize the blue shift of absorption maxima of the N-chelates (**3**–**5**) with respect to these of **1** and **2**, TDDFT calculations were performed, and the results are summarized in Tables 3 and S13.^54^ The computed UV–vis spectra (Figure S15) are in excellent agreement with the experimental ones. The broad bands of **1** and **2** are attributed exclusively to the ligand-to-metal charge transfer (LMCT) from the ligand-dominant molecular orbitals (MOs) to the Tb 4*f* orbitals (Figure S16). In comparison, the absorption maxima of **3**–**5** can be characterized as a dominant LMCT with an appreciable ligand-to-ligand charge transfer (LLCT) contribution (Figure S17). Specifically, these LLCT peaks can be assigned to ligand-dominant π → π* electronic transitions of these N-chelated ligands, leading to the blue-shifted absorption and demonstrating the stabilization of the π-orbitals upon coordination with the aromatic N-chelating ligand (Figure S18).^55^

**Figure 4:**
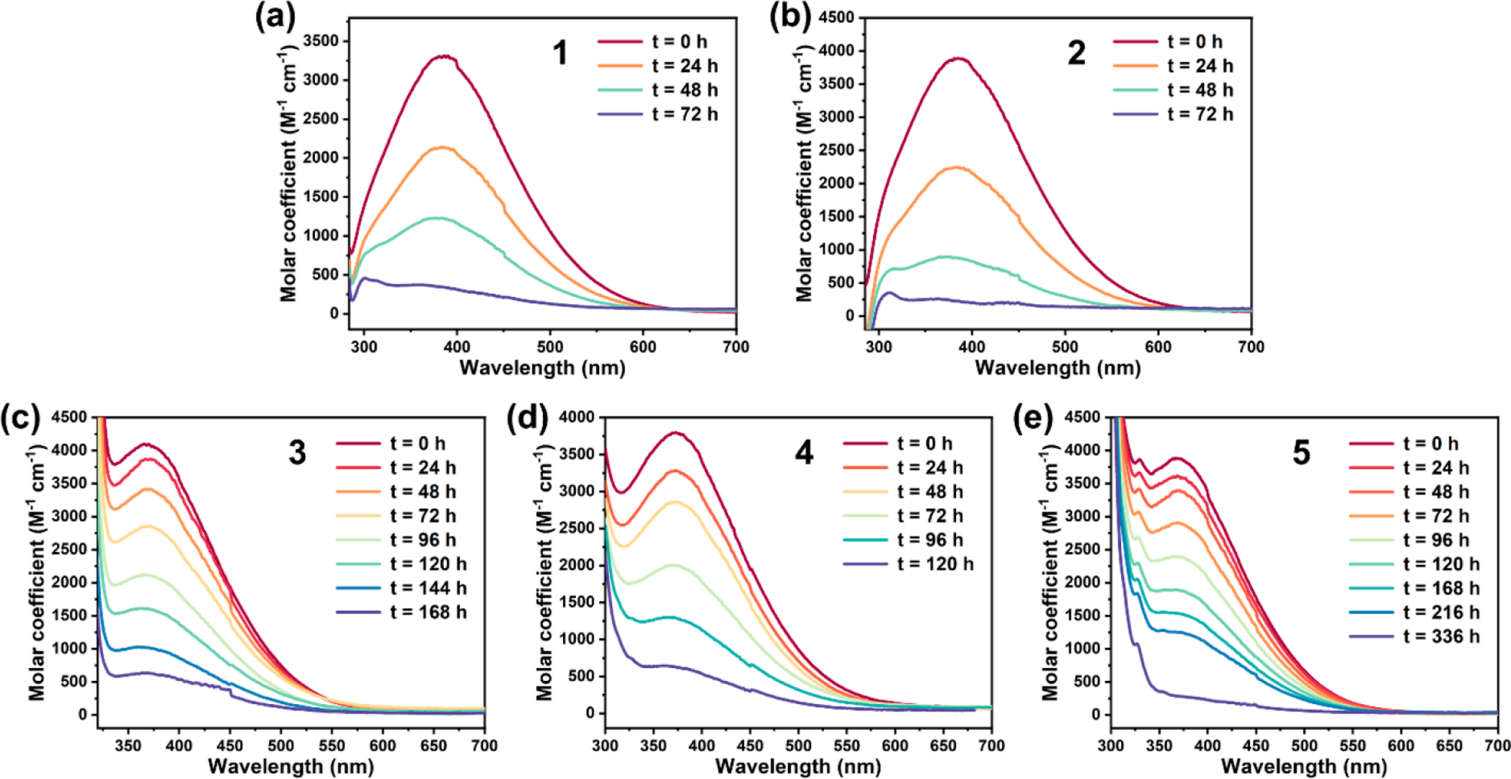
Time-dependent UV–vis spectra of **1**–**5** (a–e) in toluene at room temperature.

The solution stability of the Tb(IV) complexes was evaluated with their UV–vis spectra collected over a period of 2 weeks in toluene (Figure 4) and 1 week in dichloromethane (Figure S14) under an argon atmosphere. In toluene, the solutions of **1** and **2** decolored completely after 72 h, whereas 39%, 17%, and 49% of the characteristic absorption were retained after 120 h for the solutions of **3**–**5**, respectively. The enhancement of solution stability of **3**–**5** can be attributed to the strong intramolecular π–π interactions, as mentioned above. It should be noted that the previously reported complexes **D** and **E** survived in a 96 h UV–vis experiment;^34^ the impressive stability may be attributed, at least partly, to the shielding of the Tb(IV) center by the bulky ligands.

### DFT Calculations

The bonding interactions of the Tb(IV) center with both the siloxido and neutral ligands were studied by using DFT calculations. The theoretical and computational details are given in the SI file. The metric values of Wiberg^56^ and Mayer^57^ bond orders obtained are collected in Table S12. The average Tb-L bond orders, ranging from 0.17 to 0.25, are much smaller than those of the Tb–O_siloxido_ bonds (0.45–0.60), indicating the significant donor–acceptor character of the coordinate bond. Further analyses by EDA-NOCV^58,59^ reveal the remarkable thermostability of the N-chelates (**3**–**5**) and the tuning of the Tb-L bond interactions by the chelating ligands (Table 4). For **1** and **2**, it has been found that the donation from the occupied *sp-*hybrid orbital of the THF/DME O atom to the empty 5*d* orbitals of Tb^IV^ is dominant (23.2 kcal/mol for **1**, 20.2 kcal/mol for **2**), while the π-donation is much less significant (< ∼5 kcal/mol). The opposite is, not surprisingly, observed for the N-chelates: The N-to-Tb^IV^ π-donations are found to be 14.3, 13.3, and 11.6 kcal/mol for **3**, **4**, and **5**, respectively. This difference in π donation of the neutral ligands to Tb(IV) is presumably the determining factor in the observed stability of the N-chelates with respect to their O-chelating cognates.

**Table 4:** Energies of the σ- and π-Donation from the Neutral Ligand L to the Tb(IV) Center Produced by EDA-NOCV Analysis^a^

Energy terms	**1**	**2**	**3**	**4**	**5**
Δ*E*_*int*_	–49.90	–43.87	–56.54	–61.03	–62.56
Δ*E*_orb(L→Tb σ donation)_	–23.24	–20.15	–29.62	–21.37	–27.09
Δ*E*_orb(L→Tb π donation)_	–3.68	–4.94	–14.33	–13.26	–11.55

a All energies are given in kcal/mol.

### Magnetic Studies

**Figure 5:**
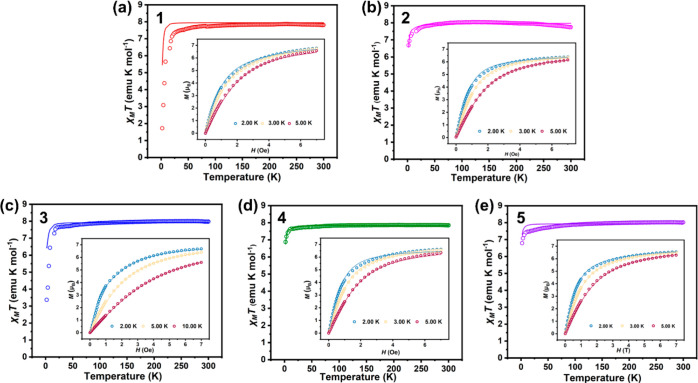
*χT* versus *T* plot of **1**–**5** (a-e) under 1000 Oe dc field. Inset: The field-dependent magnetization plots were at 2, 3, and 5 K. Solid lines are best fits with PHI.^60^

The electronic structures of the complexes were further investigated by magnetic measurements and EPR. Static magnetic susceptibilities were measured under an applied DC field of 1000 Oe with cooling from 300 to 2 K (Figure 5). The *χT* values at 300 K are 7.81, 7.75, 7.98, 7.85, and 8.02 cm^3^ K mol^–1^ for **1**–**5**, respectively, significantly smaller than the value of 11.82 cm^3^ K mol^–1^ for the mononuclear Tb(III) complex but in good agreement with the values reported for other Tb(IV) complexes.^32,36^ The *χT* decreases slowly with the lowering of temperature to ca. 20 K, at which a sudden drop occurs, reaching a minimum of 1.72, 6.68, 3.37, 6.88, and 6.79 cm^3^ K mol^–1^ for **1**–**5**, respectively. The drop in the low-temperature region is indicative of varied zero-field splitting. Field-dependent magnetizations of complexes **1**–**5** were subsequently measured at low temperatures with the field up to a maximum of 7 T (inset in Figure 5). The maximum magnetization values at 2 K and 7 T were found to be 6.77, 6.39, 6.68, 6.62, and 6.54 μ_B_ for **1**–**5**, respectively, close to the saturation magnetization value of 7 μ_B_ calculated for the 4*f*^7^ electronic configuration. The temperature- and field-dependent magnetizations were fitted with the program PHI,^60^ giving the isotropic Landé *g*-factor (*g*), axial zero-field splitting (*D*), and rhombic zero-field splitting (*E*_ZFS_) as collected in Table 5.

**Table 5:** Crystal Field Parameters of **1**–**5**

	Magnetization			EPR		
Complex	*g*	*D*	|*E*_ZFS_|	*g*	*D*	|*E*_ZFS_|
**1**	2.0067	–0.1103	0.0066	1.9958	0.2130	0.0040
**2**	2.0097	1.1484	0.0108	1.9958	0.2117	0.0043
**3**	2.0020	–0.1071	0.0084	1.9958	0.2100	0.0040
**4**	1.9953	0.7453	0.0076	1.9958	0.2050	0.0019
**5**	2.0060	0.4376	0.0020	1.9958	0.2127	0.0040

### Electron Paramagnetic Resonance Studies

**Figure 6:**
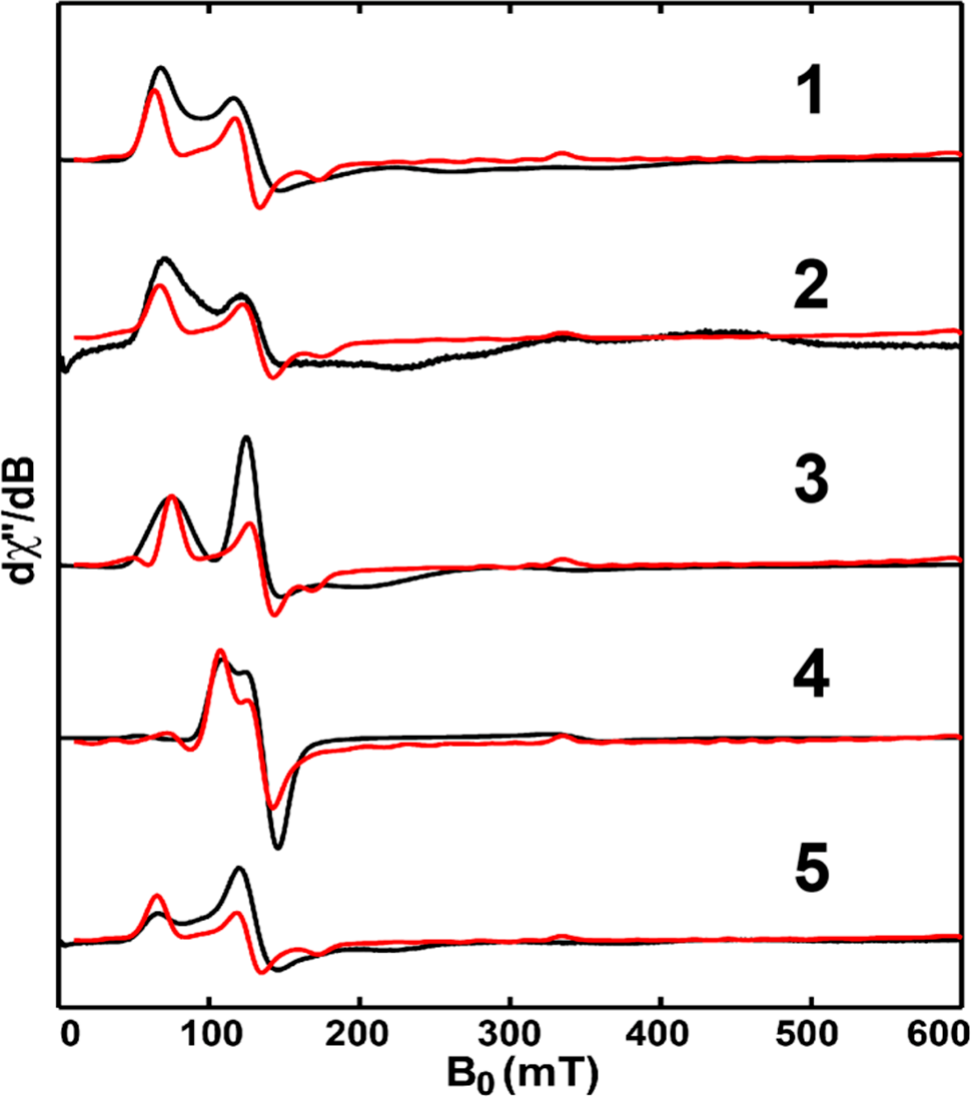
Experimental (black traces, measured at 9.36 GHz and 100 K) and simulated (red traces) X-band EPR spectra of solid **1**–**5**.

Another important technique to evaluate the *D*, *E*_ZFS_, and *g* values for magnetically active complexes is electron paramagnetic resonance (EPR) spectroscopy. The X-band (9.36 GHz) continuous-wave EPR spectra for polycrystalline samples for **1**–**5**, shown in Figure 6, were collected at 100 K. The corresponding *g* and *D* values (Table 5) were obtained via simulation.^61^ While the *g* values (1.9985) are identical for all complexes, the *D* values, all around 0.2 cm^–1^, are discernibly different. These *D*, *E*_ZFS_, and *g* values, ranging from 0.1071(22) to 1.1484(112) cm^–1^, were obtained by both fitting of the magnetization data and by EPR simulations; they are relatively small, which is consistent with the results obtained using other Tb(IV) complexes.^32,36,62^

## Conclusion

Herein, the syntheses and crystallographic structure determination of four Tb(IV) siloxido complexes with O- and N-based chelating ligands were reported together with the experimental and computational studies of their physical properties. The chelating ligands enhance the stability of the resulting complexes, as expected. More significantly, aromatic N-based chelating ligands have been found to tune effectively the electronic structures of the complexes, as evidenced by the sizable potential shifts observed for the quasi-reversible redox Tb(IV/III) process. Corresponding differences in the absorption spectra between the complexes in the comparison group provide further support for the tuning effect by the chelating ligands. The experimental findings are augmented with DFT calculations in which the ligand π-donation to the 5*d* orbitals of Tb(IV) center is primarily responsible for the stability enhancement and corresponding physical properties changes observed. The results by both fitting of the magnetization data and EPR simulations produced relatively small absolute values of zero-field splitting anticipated for a Tb(IV) ion situated in a distorted octahedral coordination geometry.

## Supporting Information Available

Materials and methods, synthesis, and characterization (PDF)
Crystal structures of 2267519 (TbPh_3_), 2267520 (**1**), 2267522 (**2**), 2267521 (**3**), 2267524 (**4**), and 2267523 (**5**) (CIF)

## Supplementary Material

pc3c00065_si_001.pdf

pc3c00065_si_002.cif
