# Supplementary material for: Tetravalent Terbium Chelates: Stability Enhancement and Property Tuning
Source: Precis Chem. 2023 Oct 2;1(10):583–91. doi: 10.1021/prechem.3c00065 (PMC11504578; doi:10.1021/prechem.3c00065)
Supplement: Supplementary file 1 — pc3c00065_si_001.pdf [file pc3c00065_si_001.pdf]

Supporting Information

Tetravalent Terbium Chelates: Stability Enhancement and Property Tuning

Tianjiao Xue,[a, c] You-Song Ding*,[a, c] Xue-Lian Jiang,[a, c] Lizhi Tao,[a] Jun Li,[a,b, c] and Zhiping Zheng*[a, c]

[a] *Department of Chemistry, Southern University of Science and Technology, Shenzhen, Guangdong 518055, China.*

[b] *Department of Chemistry and Engineering Research Center of Advanced Rare-Earth Materials of Ministry of Education, Tsinghua University, Beijing 100084, China.*

[c] *Key University Laboratory of Rare Earth Chemistry of Guangdong, Southern University of Science and Technology, Shenzhen, Guangdong 518055.*

**Corresponding authors: dingys@sustech.edu.cn; zhengzp@sustech.edu.cn.*

Contents

1. Experimental Section 3

2. Crystallographic Data 5

3. Cyclic Voltammetry 11

4. UV-Vis spectra 17

5. DFT calculations 18

6. Magnetism 23

7. Electron paramagnetic resonance (EPR) 23

8. Reference 24

1. Experimental Section

General Considerations and Methods. All manipulations were carried out with standard Schlenk techniques or in a glovebox under an argon atmosphere. Glassware was dried overnight at 120 °C before use. Toluene and n-hexane were dried over activated alumina and stored over potassium mirror before use. All other reagents including solvents were purchased from Energy-Chemical and used without further purification. Anhydrous $TbCl_3$ was synthesized by adopting a literature procedure [1]. Elemental analyses were recorded on a Carlo Erba EA1110 simultaneous CHN elemental analyzer. The UV-Vis-NIR spectra were recorded at room temperature on a UV-3600i Plus spectrophotometer in the wavelength range of 300−1600 nm with dichloromethane as reference.

$Tb(OSiPh_3)_3(THF)_3$ (**Tb^{Ph3}**). This compound was prepared by following a modified literature procedure [2]. To a solution of $[Tb(N(SiMe_3)_2)_3]$ (8.65 mmol, 5.38 g) in THF (5 mL) at room temperature was added Ph_3SiOH (25.9 mmol, 7.16 g) in THF (5 mL). The solution was intensely stirred and white product precipitated immediately with exothermic phenomenon. Supernatants were removed by filtration, and the solid product was rinsed with hexane (20 mL) and vacuum-dried (6.26 g, 60% yield).

$Tb(OSiPh_3)_4(THF)_2$ (**1**). A solution of $[N(C_6H_4Br)_3][SbCl_6]$ (0.082 g, 0.1 mmol, 1 equiv.) in MeCN (2 mL) was added at room temperature to a slurry solution of **Tb^{Ph3}** (0.12 g, 0.1 mmol, 1 equiv.) and $KOSiPh_3$ (0.03 g, 0.1 mmol, 1 equiv.) in MeCN (1 mL). The colorless suspension changed to dark blue immediately. After stirring for 2 h, bright orange precipitates were formed in a pale yellow supernatant. The orange solid was filtered and washed with MeCN (1 mL) for three times. The residue was extracted with THF (3 mL), and the dark red solution was filtered. Layering of the concentrated solution with n-hexane resulted in the dark red crystalline solid (83 mg, 51%) at -30 °C after 2 days. IR: ν [cm^{-1}] = 3063 (w), 3046 (w), 2997 (w), 2090 (w), 2879 (w), 1958 (w), 1888 (w), 1824 (w), 1772 (w), 1587 (w), 1482 (w), 1426 (m), 1185 (w), 1109 (s), 1024 (m), 997 (w), 966 (m), 905 (s), 740 (m), 696 (s). Anal. Calc. for **1**·3THF (1620.99 g mol^{-1}): $C_{92}H_{100}O_9Si_4Tb$: C, 68.40; H, 5.45; N, 0.00. Found: C, 68.41;

H, 5.12; N<0.08.

Tb(OSiPh$_3$)$_4$(DME) (**2**). As described for **1**, orange solid was obtained by a mixture of **Tb^{Ph3}**, KOSiPh$_3$ and [N(C$_6$H$_4$Br)$_3$][SbCl$_6$] in MeCN (3 mL). The residue was extracted with 1,2-dimethoxyethane (3 mL), and the dark red solution was filtered. Single crystals suitable for X-ray diffraction analysis were obtained from slow diffusion with n-hexane at -30 °C after 2 days (80 mg, 55%). IR: ν [cm^{-1}] = 3065 (w), 3048 (w), 3003 (w), 1958 (w), 1890 (w), 1828 (w), 1772 (w), 1587 (w), 1482 (w), 1426 (m), 1379 (w), 1329 (w), 1300 (w), 1257 (w), 1185 (w), 1156 (w), 1113 (m), 1024 (w), 997 (w), 900 (s), 740 (m), 696 (s). Anal. Calc. for **2**·DME (1440.72 g mol^{-1}): $C_{80}H_{80}O_8Si_4Tb$: C, 66.69; H, 5.60; N, 0.00. Found: C, 66.41; H, 5.41; N<0.07.

Tb(OSiPh$_3$)$_4$(bpy) (**3**). As described for **1**, orange solid was obtained by a mixture of **Tb^{Ph3}**, KOSiPh$_3$ and [N(C$_6$H$_4$Br)$_3$][SbCl$_6$] in MeCN (3 mL). The residue was extracted with 3 mL toluene, and the red-orange solution was filtered. A colorless solution of 2,2'-bipyridine (0.016 g, 0.1 mmol, 1 equiv.) in toluene (1 mL) was added at room temperature to the previous toluene solution of residue. After stirring for 5 minutes, the color of the solution changes from red-orange to bright orange. Single crystals suitable for X-ray diffraction analysis were obtained from concentrated toluene solution at -30 °C after 2 days(54 mg, 34%). IR: ν [cm^{-1}] = 3063 (w), 3042 (w), 2997 (w), 1958 (w), 1888 (w), 1828 (w), 1774 (w), 1597 (w), 1482 (w), 1426 (m), 1317 (w), 1185 (w), 1154 (w), 1111 (m), 1065 (w), 1041 (w), 1016 (w), 997 (w), 913 (s), 740 (m), 698 (s), 649 (w). Anal. Calc. for **3**·2tol (1600.93 g mol^{-1}): $C_{96}H_{84}N_2O_4Si_4Tb$: C, 69.52; H, 4.84; N, 1.98. Found: C, 69.57; H, 5.09; N, 1.65.

Tb(OSiPh$_3$)$_4$(bpym) (**4**). As described for **1**, orange solid was obtained by a mixture of **Tb^{Ph3}**, KOSiPh$_3$ and [N(C$_6$H$_4$Br)$_3$][SbCl$_6$] in 3 mL MeCN. The residue was extracted with 3 mL toluene, and the red-orange solution was filtered. A pale yellow solution of 2,2'-bipyrimidine (0.016 g, 0.1 mmol, 1 equiv.) in toluene (1 mL) was added at room temperature to the previous toluene solution of residue. After stirring for 5 minutes, the color of the solution changes from red-orange to bright orange. Single crystals suitable for X-ray diffraction analysis were obtained from concentrated toluene solution at -30 °C after 2 days (73 mg, 51%). IR: ν [cm^{-1}] = 3063 (w), 3042

(w), 2997 (w), 1958 (w), 1888 (w), 1828 (w), 1774 (w), 1587 (w), 1570 (w), 1552 (w), 1482 (w), 1426 (m), 1407 (m), 1331 (w), 1304 (w), 1259 (w), 1185 (w), 1111 (m), 1065 (w), 1041 (w), 1028 (w), 1012 (w), 997 (w), 913 (s), 812 (w), 740 (m), 696 (s), 651 (m), 620 (w). Anal. Calc. for **4** (1418.64 g mol^{-1}): $C_{80}H_{66}N_4O_4Si_4Tb$: C, 67.73; H, 4.69; N, 3.95. Found: C, 67.67; H, 4.63; N, 4.05.

$Tb(OSiPh_3)_4$(phen) (**5**). As described for **1**, orange solid was obtained by a mixture of **Tb^{Ph3}**, $KOSiPh_3$ and $[N(C_6H_4Br)_3][SbCl_6]$ in MeCN (3 mL). A colorless solution of 1,10-phenanthroline (0.018 g, 0.1 mmol, 1 equiv.) in toluene (1 mL) was added at room temperature to the orange solid. After stirring for 5 minutes, the color of the solution changes from red-orange to bright orange. Layering of the concentrated solution with n-hexane resulted in the red-orange crystalline solid (89 mg, 58%) at -30 °C after 2 days. IR: ν [cm^{-1}] = 3063 (w), 3046 (w), 2997 (w), 1956 (w), 1894 (w), 1824 (w), 1772 (w), 1624 (w), 1587 (w), 1519 (w), 1482 (w), 1426 (m), 1379 (w), 1331 (w), 1302 (w), 1257 (w), 1220 (w), 1185 (w), 1111 (m), 1065 (w), 1036(w), 1024 (w), 997 (w), 913 (s), 865 (m), 838 (m), 764 (w), 740 (m), 725 (w), 696 (s), 643 (w). Anal. Calc. for **5**·hex (1526.85 g mol^{-1}): $C_{90}H_{82}N_2O_4Si_4Tb$: C, 70.03; H, 4.76; N, 1.94. Found: C, 69.74; H, 4.82; N, 2.16.

2. Crystallographic Data

Single-crystal X-ray diffraction studies were carried out with the use of a Bruker D8 VENTURE diffractometer with Mo-Kα radiation (λ = 0.71073 Å) at 100 K. Using Olex2 [3], the structures were solved with the SHELXT structure solution program using Intrinsic Phasing [4] and refined with the SHELXL refinement package using Least Squares minimization [5]. All hydrogen atom positions were generated geometrically and refined with isotropic temperature factors. Cambridge Crystallographic Data Centre contains the crystal structure with the following CCDC number 2267519 (**Tb^{Ph3}**), 2267520 (**1**), 2267522 (**2**), 2267521 (**3**), 2267524 (**4**) and 2267523 (**5**).

Table S1. Collection of Crystallographic Data of **1-5** and **Tb^{Ph3}**.

	**1**	**2**	**3**	**4**	**5**	**Tb^{Ph3}**
Formula	$C_{92}H_{100}O_9Si_4Tb$	$C_{80}H_{80}O_8Si_4Tb$	$C_{96}H_{84}N_2O_4Si_4Tb$	$C_{80}H_{66}N_4O_4Si_4Tb$	$C_{90}H_{82}N_2O_4Si_4Tb$	$C_{70}H_{77}O_7Si_3Tb$
F.w.	1620.99	1440.72	1600.93	1418.64	1526.85	1273.50
T (K)	99.99	99.98	100.3	100.0	100.95	99.96
cell	monoclinic	monoclinic	monoclinic	monoclinic	orthorhombic	monoclinic
Space group	$C2/c$	$P2_1/c$	$P2_1/n$	$P2_1/n$	$P2_12_12$	$P2_1$
a (Å)	16.5145(13)	27.490(2)	20.5576(8)	13.6708(7)	16.9871(6)	14.3482(7)
b (Å)	23.0180(16)	14.4021(9)	18.3114(8)	21.3456(11)	18.5516(6)	16.4376(8)
c (Å)	21.6169(16)	18.3788(15)	23.1214(11)	23.4781(12)	12.2377(3)	14.5578(7)
α (°)	90	90	90	90	90	90
β (°)	95.653(3)	93.415(3)	112.918(2)	90.0670(10)	90	113.801(2)
γ (°)	90	90	90	90	90	90
V (Å^3)	8177.3(11)	7263.4(9)	8016.7(6)	6851.2(6)	3856.6(2)	3141.4(3)
Z	4	4	4	4	2	2
F(000)	3380.0	2980.0	3308.0	2908.0	1578.0	1320.0
D_c (g cm^{-3})	1.317	1.232	1.326	1.375	1.315	1.346
μ (mm^{-1})	0.981	1.088	0.996	6.068	1.032	1.236
Data collected/unique	42115/9415	89401/16675	70277/14097	157630/15689	62930/8855	33138/13513
$R1$ (>2σ/all data)	0.0411/0.0475	0.0542/0.0737	0.0561/0.0793	0.0202/0.0211	0.0206/0.0218	0.0327/0.0391
$wR2$ (>2σ/all data)	0.0930/0.0969	0.1131/0.1236	0.1237/0.1335	0.0580/0.0582	0.0512/0.0524	0.0677/0.0701
GOF	1.107	1.068	1.016	1.023	1.092	1.042
Residues (e Å^{-3})	1.44/-0.90	1.44/-1.72	1.89/-1.57	0.41/-0.83	1.01/-0.34	0.92/-0.88

Table S2. Selected bond lengths (Å) and angles (°) for Tb ions in **1-5** and **Tb^{Ph3}**

Complex	Tb-X bond length (Å)		X-Tb-X angle(°)			
Tb^{Ph3}	Tb2-O4	2.145(3)	O4- Tb2-O10	101.21(13)	O48- Tb2-O10	86.11(12)
	Tb2-O10	2.140(3)	O4- Tb2-O17	101.16(14)	O48- Tb2-O17	90.46(15)
	Tb2-O17	2.135(3)	O4- Tb2-O7	90.12(14)	O48- Tb2-O7	80.30(13)
	Tb2-O7	2.441(3)	O4- Tb2-O8	85.81(13)	O48- Tb2-O8	80.86(14)
	Tb2-O8	2.484(4)	O17- Tb2-O10	101.76(12)	O7- Tb2-O7	86.92(13)
	Tb2-O48	2.460(4)	O8- Tb2-O10	87.97(12)	O7- Tb2-O8	81.52(12)
1	Tb1-O4	2.079(2)	O4- Tb1-O6^{a}	81.16(8)	O4- Tb1-O5^{a}	97.63(8)
	Tb1-O4^{a}	2.079(2)	O6- Tb1-O4^{a}	81.16(8)	O5- Tb1-O4^{a}	97.63(8)
	Tb1-O5	2.043(2)	O6- Tb1-O4	82.19(8)	O6- Tb1-O5^{a}	88.94(8)
	Tb1-O5^{a}	2.043(2)	O6^{a}- Tb1-O4^{a}	82.19(8)	O5- Tb1-O6^{a}	88.94(8)
	Tb1-O6	2.400(2)	O5- Tb1-O4	97.60(8)	O5- Tb1-O5^{a}	96.02(11)
	Tb1-O6^{a}	2.400(2)	O5^{a}- Tb1-O4^{a}	97.60(8)	O6- Tb1-O6^{a}	86.10(12)
2	Tb1-O6	2.032(3)	O7- Tb1-O6	95.23(12)	O7- Tb1-O10	82.21(13)
	Tb1-O7	2.443(3)	O8- Tb1-O6	96.14(13)	O8- Tb1-O10	81.17(12)
	Tb1-O8	2.078(3)	O9- Tb1-O6	106.68(13)	O9- Tb1-O10	94.73(14)
	Tb1-O9	2.043(3)	O11- Tb1-O6	94.91(13)	O11- Tb1-O10	84.18(12)
	Tb1-O10	2.439(3)	O7- Tb1-O8	83.48(12)	O9- Tb1-O11	94.73(14)
	Tb1-O11	2.084(3)	O9- Tb1-O8	94.88(13)	O7- Tb1-O11	82.21(13)
3	Tb1-O6	2.050(4)	N1- Tb1-O6	99.39(15)	N1- Tb1-N2	65.38(14)
	Tb1-O7	2.039(4)	O8- Tb1-O6	95.54(15)	O8- Tb1-N2	84.74(14)
	Tb1-O8	2.081(4)	O9- Tb1-O6	93.82(15)	O9- Tb1-N2	82.19(14)
	Tb1-O9	2.094(4)	O7- Tb1-O6	106.19(16)	O7- Tb1-N2	89.11(16)
	Tb1-N1	2.473(4)	O8- Tb1-N1	80.99(14)	O7- Tb1-O8	95.02(15
	Tb1-N2	2.462(5)	O9- Tb1-N1	82.36(14)	O9- Tb1-O7	97.07(15)

4	Tb1-O6	2.0315(10)	N2- Tb1-O6	94.73(4)	N21- Tb1-N1	65.09(4)
	Tb1-O7	2.0694(10)	O8- Tb1-O6	100.10(4)	O8- Tb1-N1	100.08(4)
	Tb1-O8	2.0271(10)	O9- Tb1-O6	97.55(4)	O9- Tb1-N1	80.12(4)
	Tb1-O9	2.0782(10)	O7- Tb1-O6	98.03(4)	O7- Tb1-N1	79.76(4)
	Tb1-N1	2.5027(11)	O7- Tb1-N2	81.50(4)	O7- Tb1-O8	94.84(4)
	Tb1-N2	2.4967(11)	O9- Tb1-N2	81.48(4)	O9- Tb1-O8	97.95(4)
5	Tb1-O4	2.0435(18)	O4- Tb1-O4[b]	108.13(10)	N1[b]- Tb1-O4	92.67(9)
	Tb1-O4[b]	2.0435(18)	O5- Tb1-O4[b]	93.79(7)	O5- Tb1-N1	86.95(8)
	Tb1-O5	2.0851(19)	O5- Tb1-O4	94.96(7)	O5- Tb1-N1[b]	80.58(7)
	Tb1-O5[b]	2.0851(19)	O5[b]- Tb1-O4[b]	94.96(7)	O5[b]- Tb1-N1[b]	86.95(8)
	Tb1-N1	2.461(3)	O5[b]- Tb1-O4	93.79(7)	O5[b]- Tb1-N1	80.58(7)
	Tb1-N1[b]	2.461(3)	N1- Tb1-O4[b]	92.67(9)	N1[b]- Tb1-N1	66.87(14)

[a] 1-X,+Y,1/2-Z; [b] 1-X,1-Y,+Z

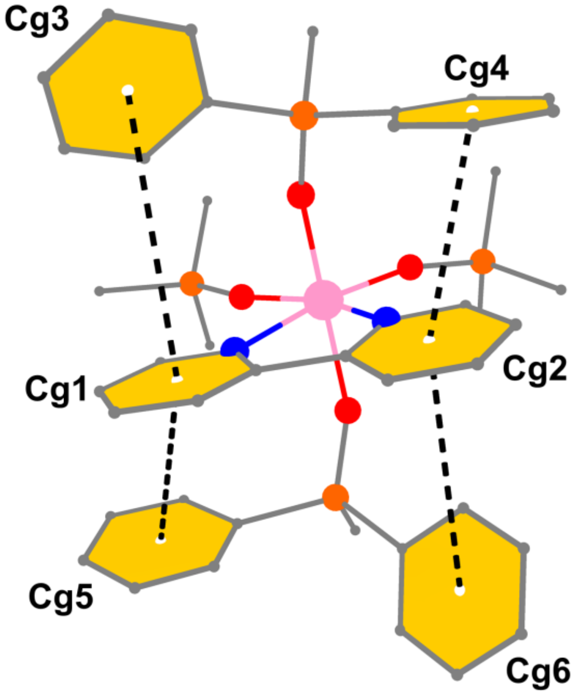

Figure S1. Illustration of the π-π stacking interaction of the phenyl group with the pyridine group of complex **3**. The yellow hexagon indicates the π-conjugate plane and the black dashed line indicates the π-π stacking interaction. The lighting effect was removed for clarity. Color code: pink (Tb), red (O), orange (Si), gray (C), blue (N).

Table S3. π-π stacking interactions in **3** (distance (Å), angles: (°)). Cg-Cg: Distance between ring centroids. Angle: Dihedral angle between planes *I* and *J*. Distance 1: Distance between plane *I* and centroid of Cg(*J*). α: Angle between Cg(*I*)-Cg(*J*) vector and normal to plane *I*. Distance 2: Distance between plane *J* and centroid of Cg(*I*). β: Angle between Cg(*J*)-Cg(*I*) vector and normal to plane *J*.

Entry	π-π interactions	Cg-Cg	Angle	Distance 1	α	Distance 2	β
1	Cg1-Cg3	4.766(4)	60.4(2)	-2.470(14)	58.78	-4.751(4)	4.55
2	Cg1-Cg5	3.617(4)	11.7(2)	3.219(6)	27.13	-3.482(5)	15.70
3	Cg2-Cg4	3.659(3)	8.94(19)	-3.266(5)	26.80	3.480(4)	17.99
4	Cg2-Cg6	4.469(3)	128.34	4.445(3)	5.94	-2.400(9)	57.52

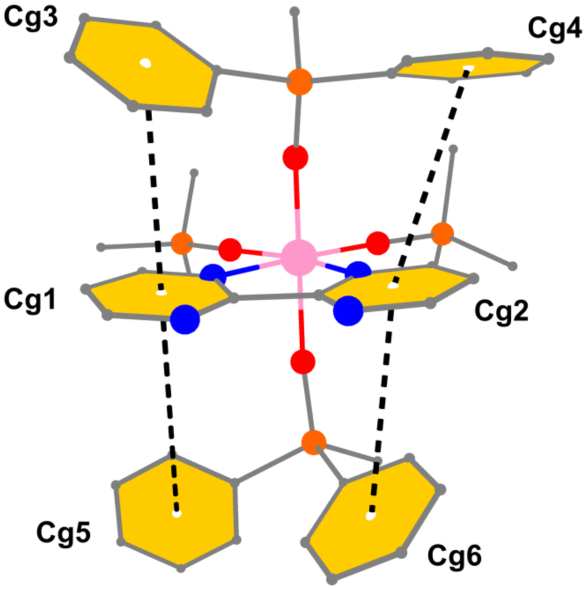

Figure S2. Illustration of the π-π stacking interaction of the phenyl group with the pyridine group of complex **4.** The yellow hexagon indicates the π-conjugate plane and the black dashed line indicates the π-π stacking interaction. The lighting effect was removed for clarity. Color code: pink (Tb), red (O), orange (Si), gray (C), blue (N).

Table S4. π-π stacking interactions in **4** (distance (Å), angles: (°))**.** Cg-Cg: Distance between ring centroids. Angle: Dihedral angle between planes *I* and *J*. Distance 1: Distance between plane *I* and centroid of Cg(*J*). α: Angle between Cg(*I*)-Cg(*J*) vector and normal to plane *I*. Distance 2: Distance between plane *J* and centroid of Cg(*I*). β: Angle between Cg(*J*)-Cg(*I*) vector and normal to plane *J*.

Entry	π-π interactions	Cg-Cg	Angle	Distance 1	α	Distance 2	β
1	Cg1-Cg3	4.0025(9)	149.06(5)	3.9655(10)	7.80	3.1395(19)	38.34
2	Cg1-Cg5	5.7883(9)	83.97(5)	-5.4157(14)	20.67	-1.463(4)	75.36
3	Cg2-Cg4	4.0683(9)	14.48(5)	3.4467(16)	32.09	-3.7993(13)	20.95
4	Cg2-Cg6	4.0279(11)	28.16(6)	-3.8772(12)	15.72	2.912(3)	43.70

Table S5. π-π stacking interactions in complex **5** (distance (Å), angles: (°)). Cg-Cg: Distance between ring centroids. Angle: Dihedral angle between planes *I* and *J*. Distance 1: Distance between plane *I* and centroid of Cg(*J*). α: Angle between Cg(*I*)-Cg(*J*) vector and normal to plane *I*. Distance 2: Distance between plane *J* and centroid of Cg(*I*). β: Angle between Cg(*J*)-Cg(*I*) vector and normal to plane *J*.

Entry	π-π interactions	Cg-Cg	Angle	Distance 1	α	Distance 2	β
1	Cg1-Cg3	4.2737(19)	49.02(12)	4.220(2)	9.09	-3.277(4)	39.93
2	Cg1-Cg5	3.523(2)	4.93(12)	-3.354(3)	17.82	3.431(2)	13.12
3	Cg2-Cg4	3.523(2)	4.93(12)	-3.354(3)	17.82	3.431(2)	13.12
4	Cg2-Cg6	4.2737(19)	49.02(12)	4.220(2)	9.09	-3.277(4)	39.93

Table S6. Continuous Shape Measures (CShM) calculations [6] for **1-5**.

Structure [ML6][a]	HP-6	PPY-6	OC-6	TPR-6	JPPY-6
**1**	33.461	25.793	0.654	14.140	29.060
**2**	31.448	27.450	1.041	15.16	31.426
**3**	31.673	26.087	1.297	14.441	30.026
**4**	30.140	26.992	1.330	15.333	29.963
**5**	31.136	26.098	1.185	13.401	30.112

[a] HP-6 = Hexagon (D_{6h}); PPY-6 = Pentagonal pyramid (C_{5v}); OC-6 = Octahedron (O_h); TPR-6 = Trigonal prism (D_{3h}); JPPY-6 = Johnson pentagonal pyramid J2 (C_{5v})

3. Cyclic Voltammetry

Cyclic Voltammetry experiments were performed using the CHI 760E Electrochemical. All experiments were performed using electrochemical cells consisting of a sealed 50 mL vial filled with Ar atmosphere, a glassy carbon (1 mm diameter) working electrode, a platinum wire counter electrode, and a silver wire for the pseudo reference electrode. The working electrode surface was polished prior to each experiment. Solutions used contained 1 mM analyte and 100 mM $[N^nBu_4][B(C_6F_5)_4]$ in dichloromethane. Ferrocene (Fc) was added as an internal standard for calibration at the end of each run.

Table S7. Electrochemical data for the redox couple of complex **1** *vs* Fc/Fc^+ in dichloromethane / $[N^nBu_4][B(C_6F_5)_4]$.

Scan rate v (mV/s)	E_{pc} (V)	E_{pa} (V)	$E°$ (V)	ΔE (V)	I_{pa}/I_{pc}
50	0.082	0.519	0.300	0.437	0.530
100	0.068	0.530	0.299	0.462	0.560
250	0.042	0.559	0.300	0.517	0.731
500	0.020	0.582	0.301	0.562	0.894
1000	-0.019	0.695	0.338	0.714	0.947

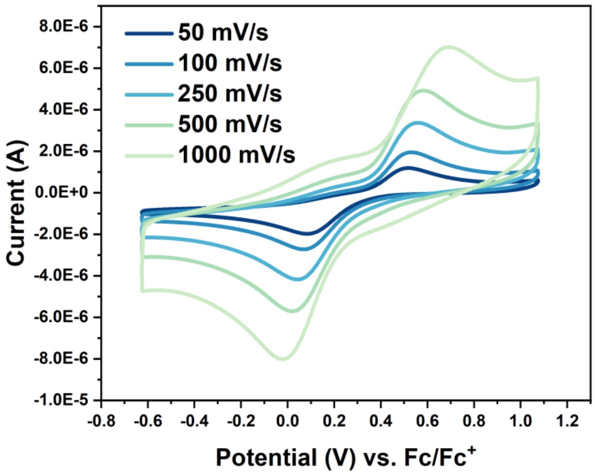

Figure S3. Cyclic voltammograms of a 1 mM solution of **1** at room temperature with varying scan rates measured in 0.1 M$[N^nBu_4][B(C_6F_5)_4]$ in dichloromethane versus Fc/Fc^+ with a glassy carbon working electrode.

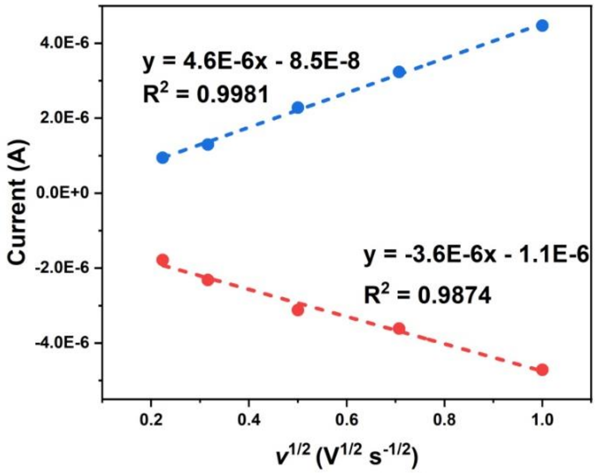

Figure S4. I_p versus $v^{1/2}$ plot of the anodic (blue) and cathodic (red) peak currents in cyclic voltammograms of **1**.

Table S8. Electrochemical data for the redox couple of complex **2** *vs* Fc/Fc^+ in dichloromethane / [N^nBu_4][$B(C_6F_5)_4$].

Scan rate v (mV/s)	E_{pc} (V)	E_{pa} (V)	$E°$ (V)	ΔE (V)	I_{pa}/I_{pc}
50	0.054	0.282	0.168	0.225	1.208
100	0.033	0.299	0.166	0.266	1.085
250	0.005	0.323	0.164	0.318	1.127
500	-0.017	0.346	0.165	0.363	0.996
1000	-0.039	0.367	0.164	0.406	1.166

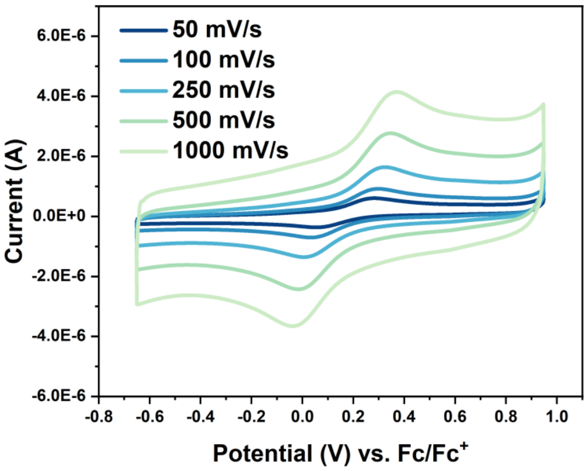

Figure S5. Cyclic voltammograms of a 1 mM solution of **2** at room temperature with varying scan rates measured in 0.1 M [N^nBu_4][$B(C_6F_5)_4$] in dichloromethane versus Fc/Fc^+ with a glassy carbon working electrode.

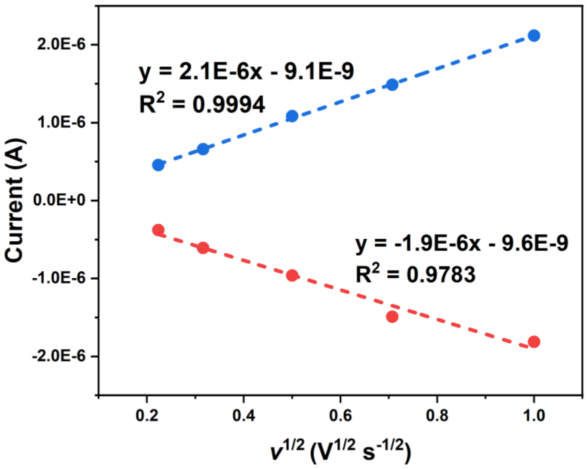

Figure S6. I_p versus $v^{1/2}$ plot of the anodic (blue) and cathodic (red) peak currents in cyclic voltammograms of **2**.

Table S9. Electrochemical data for the redox couple of complex **3** *vs* Fc/Fc^+ in dichloromethane / $[N^nBu_4][B(C_6F_5)_4]$.

Scan rate v (mV/s)	E_{pc} (V)	E_{pa} (V)	$E°$ (V)	ΔE (V)	I_{pa}/I_{pc}
50	-0.143	0.144	0.001	0.287	0.883
100	-0.118	0.172	0.027	0.290	0.868
250	-0.143	0.201	0.029	0.344	0.848
500	-0.164	0.223	0.030	0.387	0.823
1000	-0.283	0.235	-0.024	0.514	0.882

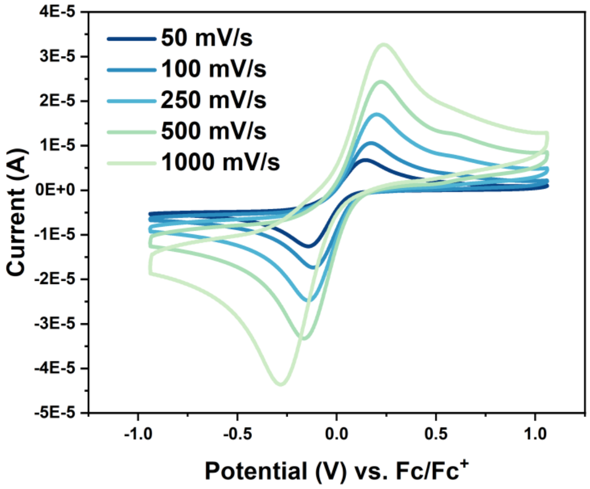

Figure S7. Cyclic voltammograms of a 1 mM solution of **3** at room temperature with varying scan rates measured in 0.1 M $[N^nBu_4][B(C_6F_5)_4]$ in dichloromethane versus Fc/Fc^+ with a glassy carbon working electrode.

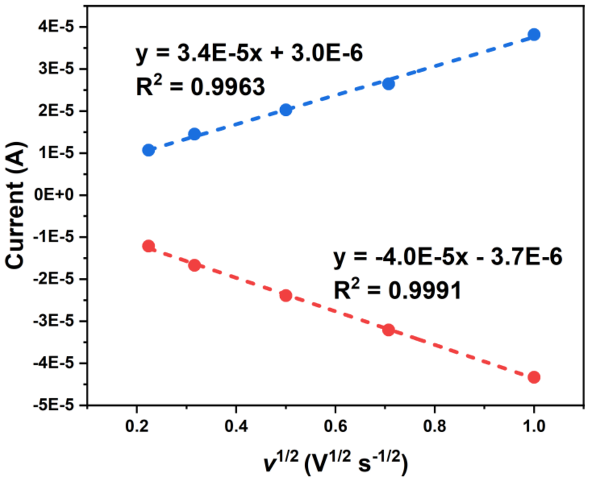

Figure S8. I_p versus $v^{1/2}$ plot of the anodic (blue) and cathodic (red) peak currents in cyclic voltammograms of **3**.

Table S10. Electrochemical data for the redox couple of complex **4** *vs* Fc/Fc$^+$ in dichloromethane / [N^{n}Bu$_4$][B(C$_6$F$_5$)$_4$].

Scan rate v (mV/s)	E_{pc} (V)	E_{pa} (V)	$E°$ (V)	ΔE (V)	I_{pa}/I_{pc}
50	-0.111	0.160	0.024	0.271	0.924
100	-0.148	0.205	0.028	0.353	0.876
250	-0.178	0.246	0.034	0.424	0.833
500	-0.208	0.266	0.029	0.474	0.792
1000	-0.236	0.286	0.025	0.522	0.831

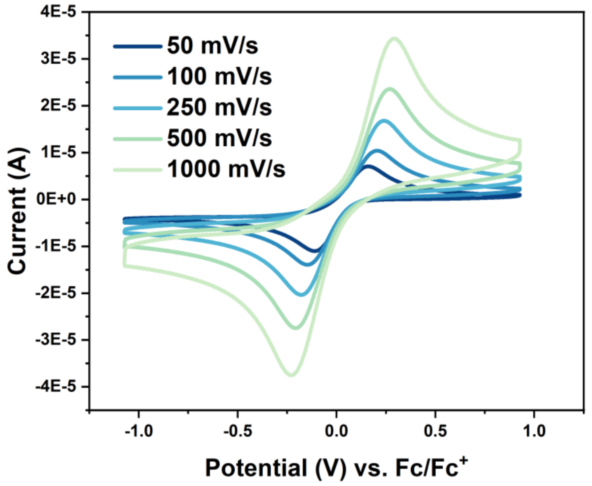

Figure S9. Cyclic voltammograms of a 1 mM solution of **4** at room temperature with varying scan rates measured in 0.1 M [N^{n}Bu$_4$][B(C$_6$F$_5$)$_4$] in dichloromethane versus Fc/Fc$^+$ with a glassy carbon working electrode.

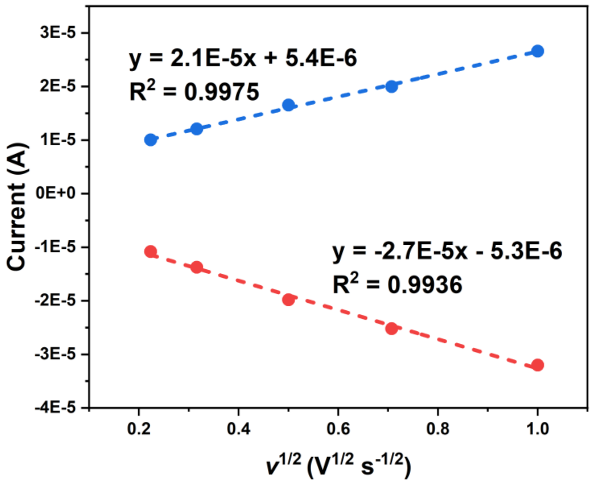

Figure S10. I_p versus $v^{1/2}$ plot of the anodic (blue) and cathodic (red) peak currents in cyclic voltammograms of **4**.

Table S11. Electrochemical data for the redox couple of complex **5** *vs* Fc/Fc^+ in dichloromethane / $[N^nBu_4][B(C_6F_5)_4]$.

Scan rate v (mV/s)	E_{pc} (V)	E_{pa} (V)	$E°$ (V)	ΔE (V)	I_{pa}/I_{pc}
50	-0.215	0.027	-0.094	0.242	1.012
100	-0.231	0.040	-0.096	0.271	1.022
250	-0.258	0.062	-0.098	0.320	1.060
500	-0.278	0.078	-0.100	0.356	1.093
1000	-0.334	0.151	-0.092	0.485	1.298

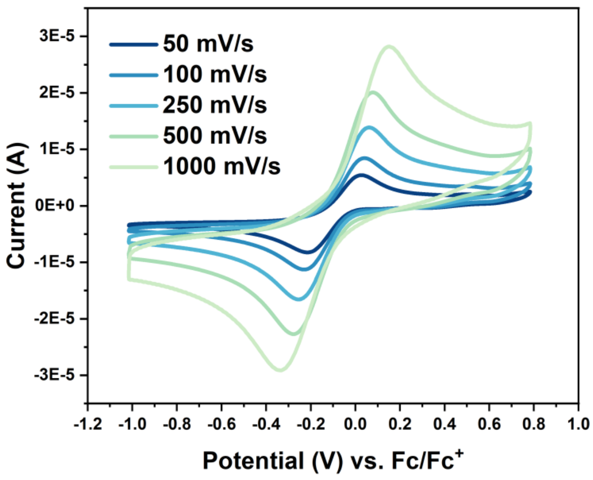

Figure S11. Cyclic voltammograms of a 1 mM solution of **5** at room temperature with varying scan rates measured in 0.1 M $[N^nBu_4][B(C_6F_5)_4]$ in dichloromethane versus Fc/Fc^+ with a glassy carbon working electrode.

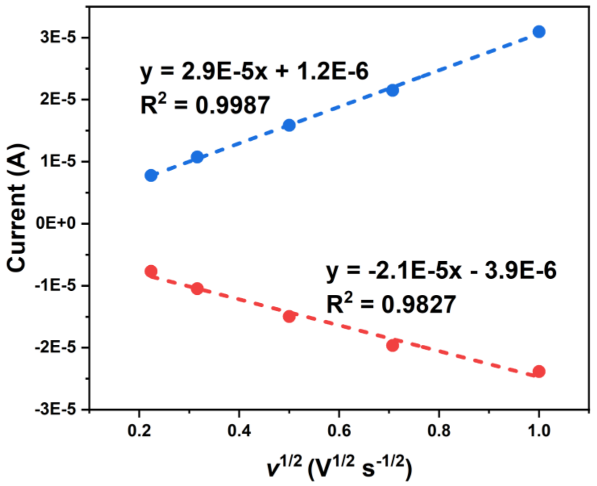

Figure S12. I_p versus $v^{1/2}$ plot of the anodic (blue) and cathodic (red) peak currents in cyclic voltammograms of **5**.

4. UV-Vis spectra

The UV-Vis spectra were recorded as 0.5 mM solutions in toluene and in dichloromethane using 1 mm cuvettes and a Perkin Elmer Lambda 365 spectrometer. To ensure reliable photophysical data and avoid decomposition, the solutions of samples were prepared and stored in the glovebox.

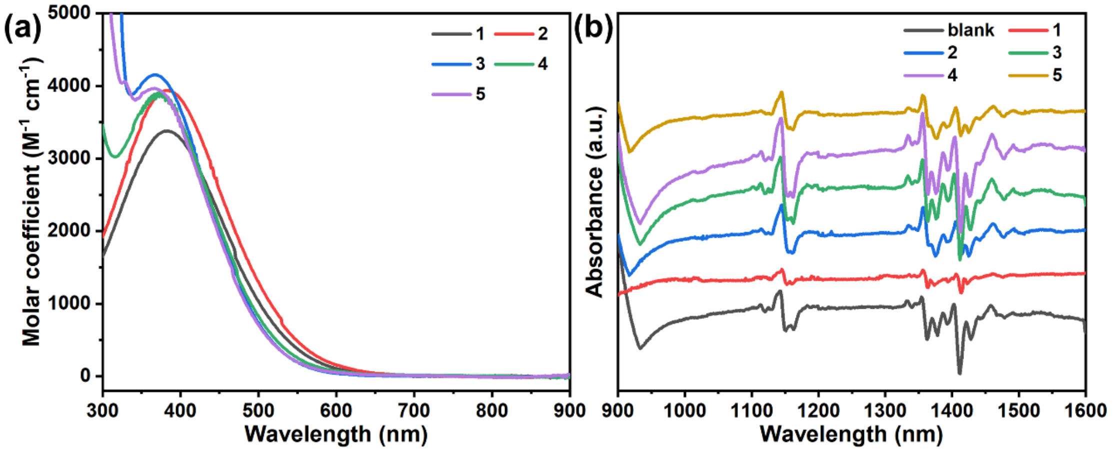

Figure S13. UV-Vis spectra of **1-5** in dichloromethane at room temperature in argon atmosphere, (a) in the 300-to-900-nm range and (b) between 900 and 1600 nm.

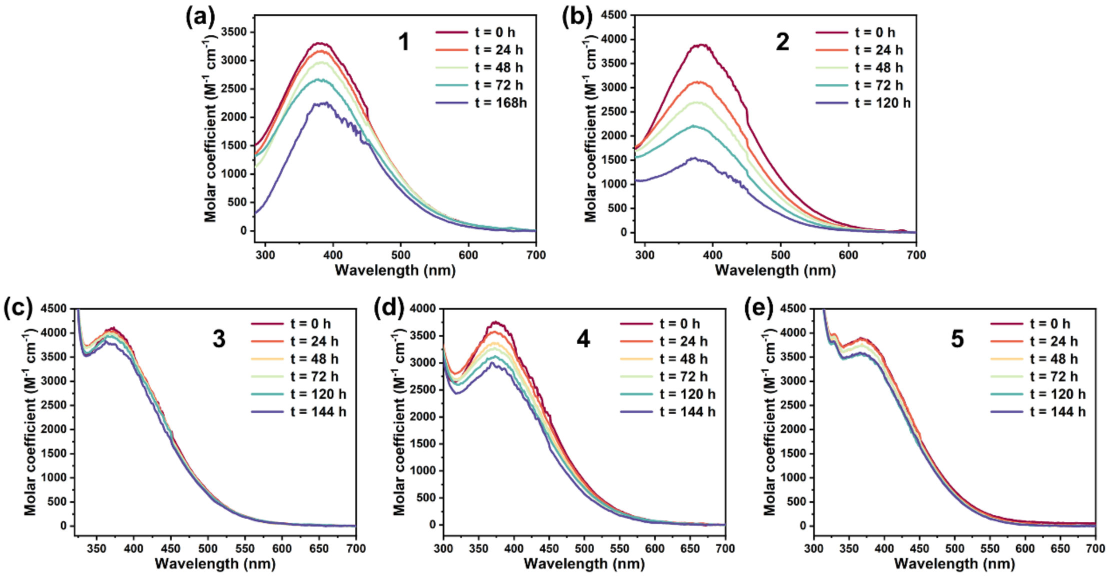

Figure S14. Time-dependent UV-Vis spectra of **1-5** (a-e) in dichloromethane at room temperature.

5. DFT calculations

All the tetravalent terbium complexes were calculated by using Density Functional Theory (DFT) at the level of hybrid B3LYP functional [7, 8], as implemented in Gaussian-16 program [9]. The geometric structures were fully optimized with default convergence criteria. Def2svp basis sets [10] was applied for C, H, N, O and Si atoms. The large-core Relativistic Effective Core Potential (RECP) [11, 12] and the associated Gaussian-type basis sets [13] developed by the Stuttgart groups was applied for Tb atom to account for the scalar relativistic (SR) effects and to save time. In order to investigate the chemical bonding between Tb and L ligands (L = THF, DME, bpy, bpym and phen for **1**-**5**, respectively), the energy decomposition analysis with natural orbitals for chemical valence (EDA-NOCV) [14, 15] was performed using ADF 2019.304 software [16]. The SR effects were taken into account with the zero-order regular approximation (ZORA) [17], while no effort was made to elucidate the effect due to spin-orbit coupling. The Wiberg [18] and Mayer [19] bond orders were calculated with Multiwfn3.7 package [20]. Time-dependent DFT (TDDFT) calculations were performed using the SAOP potential with the all-electron TZP basis set for Tb and DZP basis set for C/H/O/N/Si to simulate the electron excitation [21].

Table S12. The calculate Wiberg and Mayer bond orders for Tb-O or Tb-N bonds.*

Bond Order	**1**		**2**		**3**		**4**		**5**	
	Tb–O_{THF}	Tb–O_L	Tb–O_{DME}	Tb–O_L	Tb–N_{bpy}	Tb–O_L	Tb–N_{bpym}	Tb–O_L	Tb–N_{phen}	Tb–O_L
Wiberg	0.20	0.52	0.18	0.53	0.22	0.61	0.20	0.60	0.25	0.59
Mayer	0.19	0.45	0.17	0.46	0.22	0.53	0.20	0.55	0.25	0.52

*The bonding between Tb and L ligands (L = THF, DME, bpy, bpym, and phen for **1**-**5**) are labeled as Tb-O_{THF} for **1**, Tb-O_{DME} for **2**, Tb-N_{bpy} for **3**, Tb-N_{bpym} for **4**, and Tb-N_{phen} for **5**, respectively. The bonding between Tb and $OSiPh_3$ ligands is labeled as Tb-O_L.

Table S13. The computed positions and oscillator strengths (f) of the main peaks in the UV-Vis spectra.

Wavenumber/(nm)	f	Excitations
**Complex 1**		
388	0.0069	L → Tb(4f)
394	0.0051	L → Tb(4f)
**Complex 2**		
384	0.0091	L → Tb(4f)
377	0.0065	L → Tb(4f)
**Complex 3**		
378	0.0055	L → N-chelated ligands (π*)
377	0.0028	L → Tb(4f)
376	0.0067	L → Tb(4f)
373	0.0107	L → Tb(4f)
**Complex 4**		
378	0.0017	L → N-chelated ligands (π*)
377	0.0031	L → N-chelated ligands (π*)
375	0.0100	L → Tb(4f)
373	0.0071	L → Tb(4f)
372	0.0066	L → Tb(4f)
**Complex 5**		
378	0.0013	L → N-chelated ligands (π*)
376	0.0032	L → N-chelated ligands (π*)
374	0.0105	L → Tb(4f)
371	0.0029	L → Tb(4f)

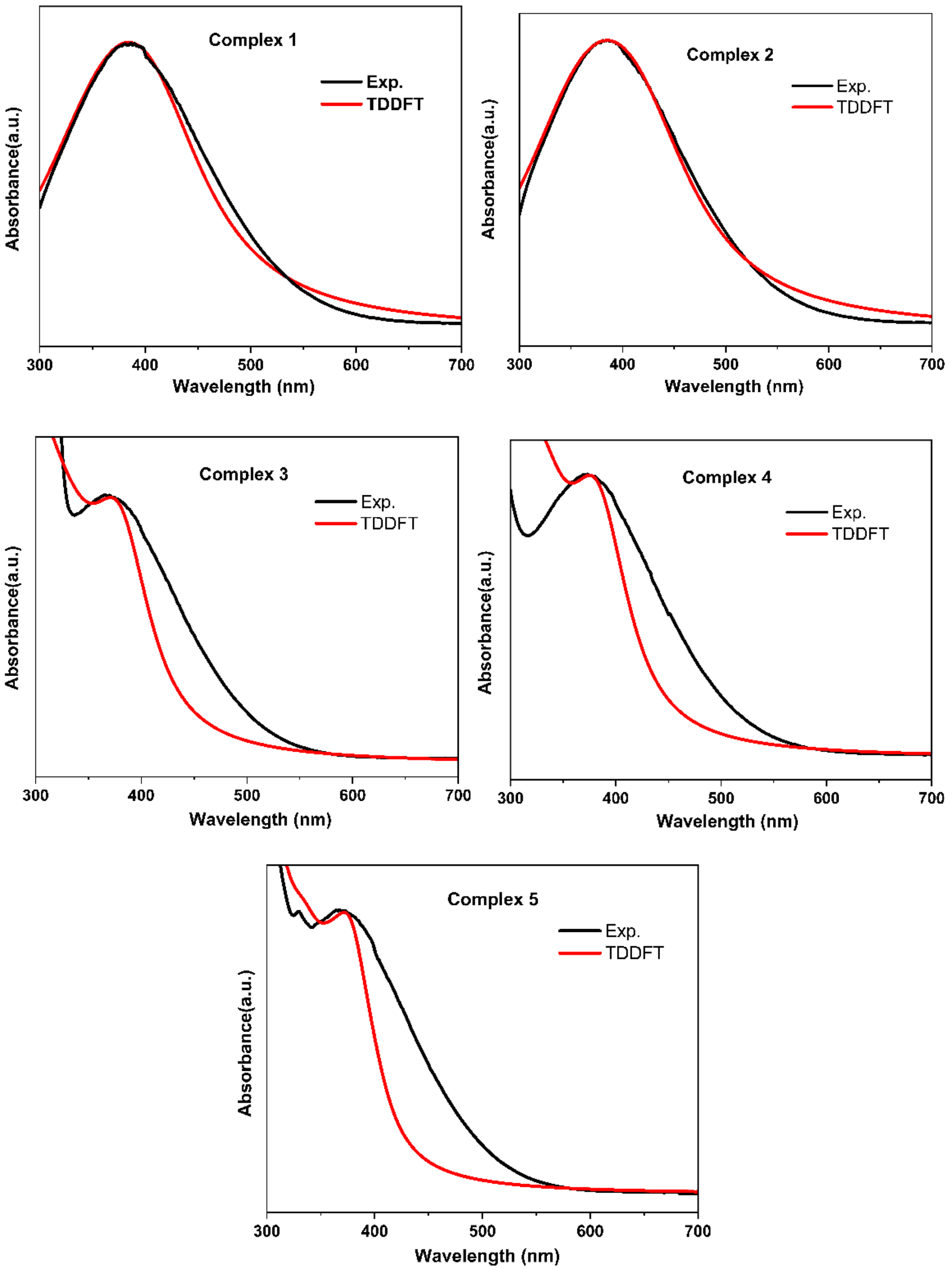

Figure S15. The simulated (red) and experimental (black) UV-Vis absorption spectra of complexes **1-5**.

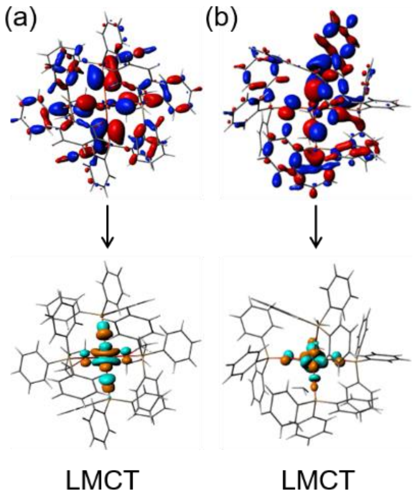

Figure S16. The dominant electronic excitations of ligand-to-metal charge transfer (LMCT) in the absorption maxima for complexes (a) **1** and (b) **2**.

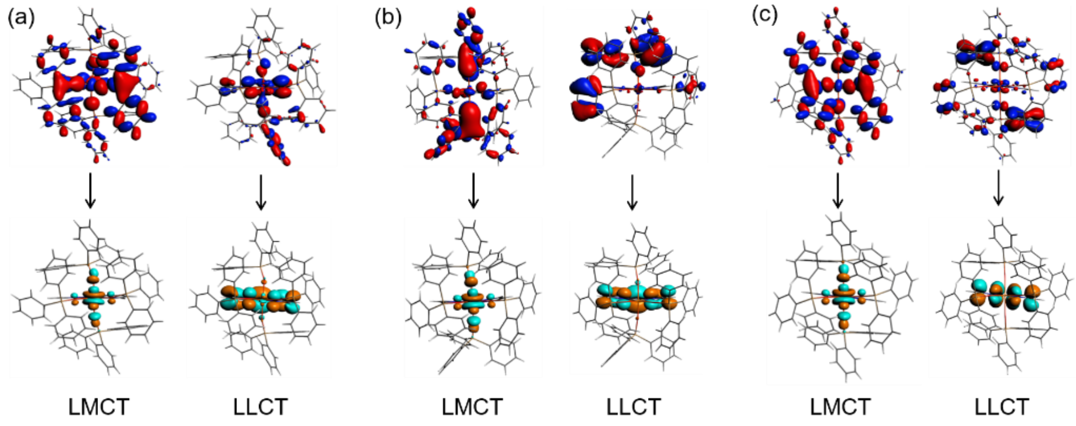

Figure S17. The dominant electronic excitations of ligand-to-metal charge transfer (LMCT) and ligand-to-ligand charge transfer (LLCT) in the absorption maxima for complexes (a) **3**, (b) **4**, and (c) **5.**

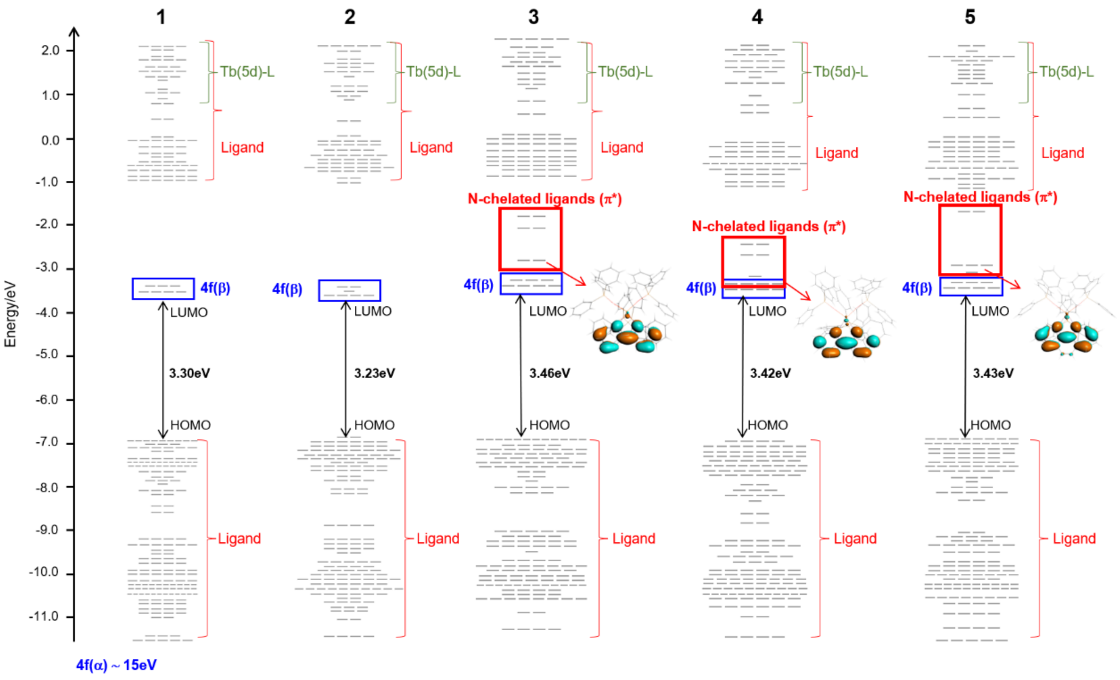

Figure S18. Combined (α+β) molecular orbital (MO) diagrams of complexes **1-5**. The MO lines in the blue box represent the Tb-4f AOs in the MOs. The red-boxed MO lines are from the *N*-chelating ligands.

6. Magnetism

Magnetic susceptibility measurements were carried out with a Quantum Design MPMS-3 SQUID magnetometer, with polycrystalline samples sealed with melted eicosane in NMR tubes under a vacuum. Static magnetic susceptibilities were measured under an applied DC field of 1000 Oe upon cooling from 300 K to 2 K. Field-dependent magnetizations of complexes **1-5** were measured at low temperatures with field up to a maximum field of 7 T. The temperature and field dependent magnetizations for **1-5** were fitted with the program PHI to extract the isotropic Landé g-factor (g), axial zero-field splitting (D) and rhombic zero-field splitting (E) [22].

7. Electron paramagnetic resonance (EPR)

Electron paramagnetic resonance (EPR) spectra were collected with a Bruker EMXplus EMXmicro EPR at 100 K and 9.8 GHz, followed by simulations with EasySpin [23], using poly-crystalline sample for **1-5**.

8. Reference

[1] Huang, W.; Upton, B. M.; Khan, S. I.; Diaconescu, P. L., Synthesis and characterization of paramagnetic lanthanide benzyl complexes. *Organometallics* **2013,** *32*, 1379-1386.

[2] McGeary, M. J.; Coan, P. S.; Folting, K.; Streib, W. E.; Caulton, K. G., Yttrium and lanthanum silyloxide complexes. *Inorg. Chem.* **1991,** *30*, 1723-1735.

[3] Dolomanov, O. V.; Bourhis, L. J.; Gildea, R. J.; Howard, J. A. K.; Puschmann, H., OLEX2: a complete structure solution, refinement and analysis program. *J. Appl. Crystallogr.* **2009,** *42*, 339-341.

[4] Sheldrick, G., SHELXT - Integrated space-group and crystal-structure determination. *Acta Crystallogr. Sect. A* **2015,** *71*, 3-8.

[5] Sheldrick, G., Crystal structure refinement with SHELXL. *Acta Crystallogr. Sect. C* **2015,** *71*, 3-8.

[6] Alvarez, S.; Avnir, D.; Llunell, M.; Pinsky, M., Continuous symmetry maps and shape classification. The case of six-coordinated metal compounds. *New J. Chem.* **2002,** *26*, 996-1009.

[7] Becke, A. D., Density-functional exchange-energy approximation with correct asymptotic behavior. *Phys. Rev. A* **1988,** *38*, 3098-3100.

[8] Lee, C.; Yang, W.; Parr, R. G., Development of the Colle-Salvetti correlation-energy formula into a functional of the electron density. *Phys. Rev. B* **1988,** *37*, 785-789.

[9] Frisch, M.; Trucks, G.; Schlegel, H.; Scuseria, G.; Robb, M.; Cheeseman, J.; Scalmani, G.; Barone, V.; Petersson, G.; Nakatsuji, H., Gaussian 16. In *Revision A*, Gaussian, Inc., Wallingford CT: 2016; Vol. 3.

[10] Weigend, F.; Ahlrichs, R. Balanced Basis Sets of Split Valence, Triple zeta valence and quadruple zeta valence quality for H to Rn: Design and assessment of accuracy. *Phys. Chem. Chem. Phys.* **2005**, *7*, 3297−3305.

[11] Dolg, M.; Stoll, H.; Savin, A.; Preuss, H., Energy-adjusted pseudopotentials for the rare earth elements. *Theor. Chim. Acta* **1989,** *75*, 173-194.

[12] Dolg, M.; Stoll, H.; Preuss, H., A combination of quasirelativistic pseudopotential and ligand field calculations for lanthanoid compounds. *Theor. Chim. Acta* **1993,** *85*,

441-450.
[13] Maron, L.; Eisenstein, O., Do f electrons play a role in the lanthanide−ligand bonds? A DFT study of $Ln(NR_2)_3$; R = H, SiH_3. *J. Phys. Chem. A* **2000,** *104*, 7140-7143.
[14] Michalak, A.; Mitoraj, M.; Ziegler, T., Bond orbitals from chemical valence theory. *J. Phys. Chem. A.* **2008,** *112*, 1933-1939.
[15] Mitoraj, M. P.; Michalak, A.; Ziegler, T., A combined charge and energy decomposition scheme for bond analysis. *J. Chem. Theory Comput.* **2009,** *5*, 962-975.
[16] te Velde, G.; Bickelhaupt, F. M.; Baerends, E. J.; Fonseca Guerra, C.; van Gisbergen, S. J. A.; Snijders, J. G.; Ziegler, T., Chemistry with ADF. *J. Comput. Chem.* **2001,** *22*, 931-967.
[17] van Lenthe, E.; van Leeuwen, R.; Baerends, E. J.; Snijders, J. G., Relativistic regular two-component Hamiltonians. *Int. J. Quantum Chem.* **1996,** *57*, 281-293.
[18] Wiberg, K. B., Application of the pople-santry-segal CNDO method to the cyclopropylcarbinyl and cyclobutyl cation and to bicyclobutane. *Tetrahedron* **1968,** *24*, 1083-1096.
[19] Mayer, I., Charge, bond order and valence in the AB initio SCF theory. *Chem. Phys. Lett*. **1983,** *97*, 270-274.
[20] Lu, T.; Chen, F., Multiwfn: A multifunctional wavefunction analyzer. *J. Comput. Chem.* **2012,** *33*, 580-592.
[21] Gritsenko, O. V.; Schipper, P. R. T.; Baerends, E. J., Approximation of the exchange-correlation Kohn–Sham potential with a statistical average of different orbital model potentials. *Chem. Phys. Lett.* **1999,** *302*, 199-207.
[22] Chilton, N. F.; Anderson, R. P.; Turner, L. D.; Soncini, A.; Murray, K. S., PHI: A powerful new program for the analysis of anisotropic monomeric and exchange-coupled polynuclear d- and f-block complexes. *J Comput Chem.* **2013,** *34*, 1164-1175.
[23] Stoll, S.; Schweiger, A., EasySpin, a comprehensive software package for spectral simulation and analysis in EPR. *J. Magn. Reson.* **2006,** *178*, 42-55.
